# CD31^+^ Cell Enrichment Enhances Therapeutic Effects of Stromal Vascular Fraction in Experimental Primary Osteoarthritis: A Preclinical Study in the Dunkin Hartley Guinea Pig Model

**DOI:** 10.1002/advs.76187

**Published:** 2026-07-11

**Authors:** Sijia Zhou, Tobias Winkler, Bin Chen, Marta Grzeski, Yilong Yang, Florian N. Fleckenstein, Dominik Geisel, Alexander Hildebrandt, Daniela Aschenbrenner, Marie Agatha Mohn, Benjamin Claass, January Weiner, Sven Geissler, Georg N. Duda, Søren P. Sheikh, Oliver Klein, Tazio Maleitzke

**Affiliations:** ^1^ Julius Wolff Institute Berlin Institute of Health at Charité – Universitätsmedizin Berlin Berlin Germany; ^2^ BIH Center for Regenerative Therapies Berlin Institute of Health at Charité – Universitätsmedizin Berlin Berlin Germany; ^3^ Center for Musculoskeletal Surgery Charité – Universitätsmedizin Berlin Corporate Member of Freie Universität Berlin and Humboldt‐Universität zu Berlin Berlin Germany; ^4^ Department of Bone and Joint Surgery Department of Orthopedics Renji Hospital School of Medicine Shanghai Jiao Tong University Shanghai China; ^5^ Imaging Mass Spectrometry Unit Berlin Institute of Health at Charité – Universitätsmedizin Berlin Berlin Germany; ^6^ Department of Diagnostic and Interventional Radiology Charité – Universitätsmedizin Berlin Corporate Member of Freie Universität Berlin and Humboldt‐Universität zu Berlin Berlin Germany; ^7^ Blue Cell Therapeutics Copenhagen Denmark; ^8^ Core Unit Bioinformatics Berlin Institute of Health at Charité – Universitätsmedizin Berlin Berlin Germany; ^9^ Trauma Orthopaedic Research Copenhagen Hvidovre (TORCH) Department of Orthopaedic Surgery Copenhagen University Hospital‐Amager and Hvidovre Hvidovre Denmark; ^10^ Department of Clinical Medicine University of Copenhagen Copenhagen Denmark

**Keywords:** cartilage regeneration, cell therapy, extracellular matrix remodeling, immunomodulation, inflammation, stromal vascular fraction

## Abstract

Despite the growing burden of osteoarthritis (OA), effective disease‐modifying treatments remain limited. Hyaluronic acid, platelet‐rich plasma, and hydrogels are in clinical use, but their efficacy beyond placebo is debated. Cell‐based therapies are continuously investigated for their disease‐modifying potential. Stromal vascular fraction (SVF), a minimally processed and clinically accessible cell product, has shown therapeutic potential, but remains poorly characterized in respect to its pro‐regenerative cell types and molecular signaling. To determine whether CD31^+^ cell enrichment enhances the regenerative efficacy of SVF, a comparative efficacy study was conducted using the Dunkin Hartley guinea pig model of spontaneous OA. Spatial proteomics reveals that both treatments modulate cartilage extracellular matrix (ECM) composition, reducing fibronectin and COL1A1 levels, with CD31^+^ SVF showing more pronounced effects. Both therapies attenuate cartilage fibrosis, increase aggrecan, suppress MMP‐13, and reduce TNF‐α and MCP‐1 expression. Compared to SVF, CD31^+^ SVF shows greater and more consistent improvements in subchondral and trabecular bone integrity. These findings demonstrate that CD31^+^ enrichment enhances the therapeutic potential of SVF, likely through more consistent modulation of ECM remodeling and osteochondral integrity. Our results provide a preclinical rationale for the testing of CD31^+^‐enriched SVF as a biologically refined cell therapy approach for OA, with potential disease modifying properties.

## Graphical abstract

Both SVF and CD31^+^ SVF improve spontaneous osteoarthritis, but CD31^+^ enrichment refines the therapeutic profile rather than broadly enhancing all effects. More distinct benefits are observed in extracellular matrix remodeling, osteochondral integrity, and subchondral/trabecular bone preservation, supporting CD31^+^‐enriched SVF as a biologically enhanced strategy for osteoarthritis treatment.

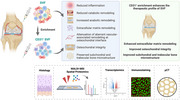

## 1 Introduction

Osteoarthritis (OA) is a highly prevalent chronic joint disease where the global burden continues to rise with population ageing, obesity, and sedentary lifestyles, placing substantial pressure on healthcare systems worldwide [1]. Globally, OA affects more than 500 million people, with knee OA (KOA) accounting for the largest share of OA‐related disability and disease burden [2]. A central challenge in OA management is the limited intrinsic regenerative capacity of articular cartilage, which is further complicated by the complex multi‐tissue nature of the disease, including progressive cartilage degradation, subchondral bone remodeling, synovial inflammation, and infrapatellar fat pad (IFP) fibrosis [3]. Moreover, OA is increasingly recognized as a heterogeneous disease comprising multiple molecular endotypes, that is, biologically distinct subsets driven by different pathophysiological mechanisms [4]. This heterogeneity underscores the need for therapies capable of acting across several pathological axes rather than targeting a single mechanism. Although a range of symptomatic treatments, including oral supplements, intra‐articular (IA) hyaluronic acid, platelet‐rich plasma, and hydrogels, are used in clinical practice to alleviate pain and reduce inflammation, no therapy has yet received regulatory approval as a disease‐modifying osteoarthritis drug (DMOAD) [5]. In this context, cell‐based strategies have emerged as a regenerative approach, continuously investigated for the potential to influence the structural course of OA. Clinical translation remains constrained by limited accessibility, stringent manufacturing requirements for Good Manufacturing Practices (GMP)‐compliant products, complex regulatory pathways, and conflicting evidence on efficacy [6, 7, 8, 9]. In the current OA research and translational landscape, stromal vascular fraction (SVF)‐based therapy is being explored as an intermediate biologic option for patients who remain inadequately controlled with conservative treatments but are not yet candidates for joint replacement surgery, thereby potentially addressing an important therapeutic gap in moderate‐stage disease [10].

SVF is a heterogeneous cell population derived from adipose tissue, comprising immune, endothelial, and progenitor cells [11]. SVF isolation can be achieved through either mechanical or enzymatic processing. Mechanical methods avoid exogenous enzymes and may better align with simplified processing workflows and some minimal‐manipulation frameworks [12]. In contrast, enzymatic digestion can provide more complete stromal tissue dissociation, higher recovery of viable nucleated cells, and a more thoroughly characterized SVF fraction [12]. This is particularly advantageous when analyzing or enriching defined cellular subsets. Unlike mesenchymal stromal cells (MSCs), SVF is easily accessible and requires minimal processing before clinical application. It has been shown to exhibit anti‐inflammatory and immunomodulatory properties [13, 14, 15] and can be applied as a point‐of‐care therapy, without the need for further in vitro expansion [11]. The concept circumvents current limitations associated with advanced cellular products, that include expansion‐related senescence, scalability, and manufacturing delays that may impair cell functionality or treatment availability [16, 17]. However, the heterogeneous composition of SVF is associated with both potential advantages and limitations, as cellular diversity may enable broad paracrine signaling but also make the therapeutic product difficult to characterize [18, 19]. Enrichment of functionally relevant SVF subpopulations is crucial to improve therapeutic consistency and narrow down biological activity.

Among the cellular constituents of SVF, CD31^+^ cells, comprising endothelial progenitor cells, monocytes/macrophages, and subsets of T and B lymphocytes, are particularly relevant immunomodulators [19, 20]. In vitro and in vivo data show that CD31^+^ cells modulate T‐cell‐mediated immune responses and help maintain immune balance, thereby contributing to inflammation resolution under pathological conditions [20, 21, 22]. Previous work from our group demonstrated that CD31^+^ cells isolated from peripheral blood facilitate bone regeneration under biologically impaired conditions through combined immunomodulatory and tissue‐regulatory mechanisms, including enhanced secretion of the anti‐inflammatory cytokine IL‐1 receptor antagonist (IL‐1RA), suppression of pro‐inflammatory mediators such as GM‐CSF and IFN‐γ, support of vascular homeostasis, and reduction of tissue fibrosis [20]. Notably, both the enrichment and depletion of CD31^+^/CD14^+^ monocytes from the mixed CD31^+^ population diminished tissue regeneration, suggesting that the therapeutic effect depends on the coordinated action of multiple CD31^+^ subsets rather than a single cell type [20]. Complementary findings from adipose tissue‐derived SVF further support the view that CD31^+^ cells are a biologically active subpopulation with vascular‐regulatory potential [19]. CD31 is a defining marker of type H vessels, a specialized vascular subtype associated with osteogenesis and bone remodeling [23]. Abnormal vascular remodeling at the osteochondral interface is increasingly recognized as a contributor to OA progression [24]. Given that OA is characterized by concurrent cartilage degradation, chronic low‐grade inflammation, and pathological subchondral bone remodeling, CD31^+^ cells may influence multiple pathological axes relevant to the disease through their combined immunomodulatory, anti‐fibrotic, microvascular‐regulatory, and osteoregulatory properties.

Accordingly, the present study was designed to address three central questions: (i) whether a single IA injection of SVF can attenuate the progression of spontaneous primary KOA, (ii) whether CD31^+^ cell enrichment enhances the therapeutic efficacy of SVF relative to unfractionated SVF, and (iii) which molecular changes and candidate pathways are associated with cartilage‐protective, anti‐inflammatory, and osteochondral remodeling effects of both cell products. We hypothesized that SVF would attenuate the progression of spontaneous primary OA and that enrichment of the CD31^+^ fraction would refine its therapeutic profile by enhancing selected effects on cartilage preservation, inflammation control, and osteochondral remodeling. To test this hypothesis, we employed the DH guinea pig model, in which spontaneous, age‐dependent KOA that recapitulates key features of human primary disease, including cartilage degeneration, subchondral bone remodeling, and synovial inflammation, develops, without requiring surgical or chemical induction [25]. Using this model, a comprehensive multimodal analytical framework integrating histological, molecular, imaging, and spatial proteomic approaches was applied to assess cartilage degeneration, ECM remodeling, inflammatory activity, osteochondral integrity, and treatment safety.

## 2 Results

### 2.1 Age‐Dependent Primary OA in the DH Guinea Pig Model

**FIGURE 1:**
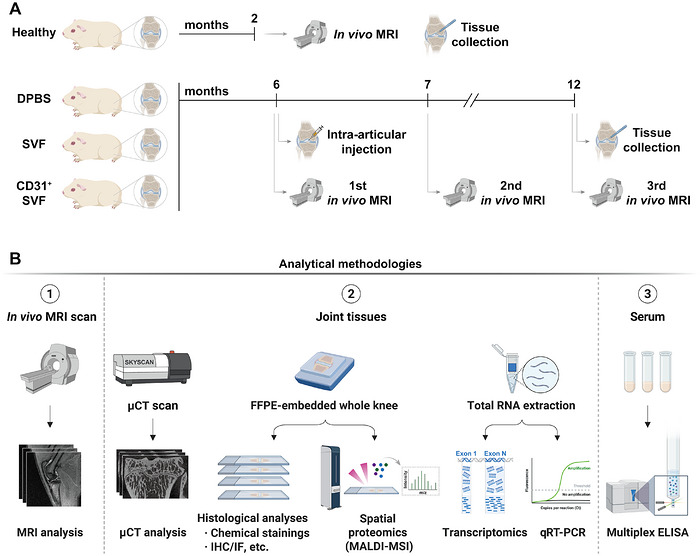
Experimental design and analytical methodologies. (A) Schematic diagram illustrating the study design. (B) Overview of analytical methodologies, including magnetic resonance imaging (MRI), microcomputed tomography (µCT), histology, matrix‐assisted laser desorption/ionization mass spectrometry imaging (MALDI‐MSI), transcriptomics, real‐time quantitative polymerase chain reaction (RT‐qPCR), and multiplex enzyme‐linked immunosorbent assay (ELISA).

To validate the DH guinea pig as a spontaneous KOA model [25], we first compared ten animals that received IA Dulbecco's phosphate‐buffered saline (DPBS) at the age of 6 months and who were euthanized at 12 months (DPBS group) to ten 2‐month‐old healthy animals (healthy group) for further analyses (Figure 1A,B). As 2‐month‐old DH guinea pigs are still skeletally immature, this group was used as a young healthy reference rather than a fully mature age‐matched normal control. Expanded healthy‐reference data for additional cartilage‐ and inflammation‐related markers are provided in Figures S4 and S5. Comparisons confirmed the age‐dependent progressive OA pathology in this model.

**FIGURE 2:**
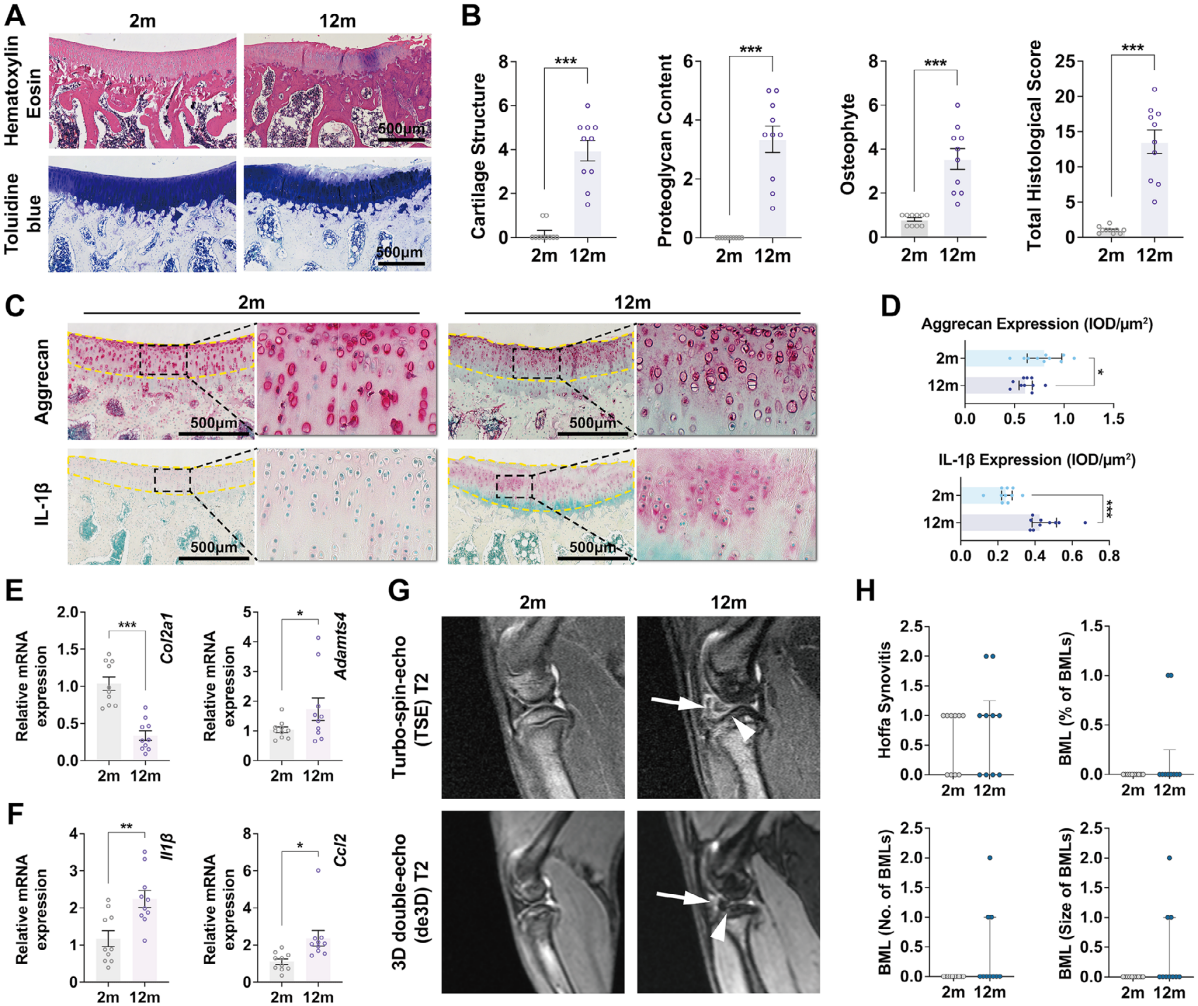
Progressive cartilage degeneration and inflammation in the 12‐month‐old DPBS group compared to the 2‐month‐old healthy control group in DH guinea pigs. (A) Representative H&E‐ and TB‐stained sections of medial tibial cartilage. (B) OARSI histological scoring of tibial cartilage in the healthy (analyzed at 2 months) and DPBS group (analyzed at 12 months). (C and D) Immunohistochemical staining and analysis (IOD) of OA‐related markers, including aggrecan and IL‐1β, in tibial cartilage. Yellow dashed lines in (C) outline the region of interest (ROI) for analysis. (E and F) mRNA expression analysis of *Col2a1* and *Adamts4* in tibial cartilage (E), and *Il1β* and *Ccl2* in the IFP (F). (G) Representative MRI images highlighting differences in Hoffa‐synovitis (arrows) and overall femoral and tibial bone marrow lesions (arrowheads). (H) Modified MOAKS scores based on in vivo MRI in the healthy control and DPBS animals to evaluate joint pathology. Individual data points are presented as means ± SEM (B, D, E, F, and H). Statistical comparisons were performed using two‐way ANOVA. ^*^ *p* < 0.05, ^**^ *p* < 0.01, and ^***^ *p* < 0.001.

Histological staining with Hematoxylin and eosin (H&E) and Toluidine blue (TB) of the medial tibial cartilage showed more severe cartilage destruction in the older DPBS group compared to the healthy control group (Figure 2A). Quantitative assessment using Osteoarthritis Research Society International (OARSI) scoring [26] based on TB staining demonstrated significantly higher scores in the DPBS group compared to the healthy control group across multiple parameters, including cartilage structure, proteoglycan content, osteophyte formation, and total histological scores (Figure 2B).

To further evaluate OA‐related molecular changes, histomorphometric analysis of tibial cartilage was conducted. Measurement of integrated optical density (IOD) showed a significant reduction in aggrecan expression in the DPBS group compared to the healthy control group (*p* = 0.025). For inflammatory activity, the older DPBS group exhibited markedly elevated expression of interleukin (IL)‐1β (IOD: *p* < 0.001) (Figure 2C,D). These findings indicate increased cartilage degeneration and inflammatory activity in the DPBS group relative to the young healthy control.

This was next confirmed at the mRNA expression level. Tibial cartilage of the DPBS group showed significantly lower alpha‐1 chain of type II collagen (*Col2a1*) expression (*p* < 0.001) and higher a disintegrin and metalloproteinase with thrombospondin motifs (*Adamts) 4* expression (*p* = 0.048) compared to the healthy control group (Figure 2E). Likewise, the IFP exhibited elevated *Il1β* (*p* = 0.004) and C‐C motif ligand 2 (*Ccl2*) (*p* = 0.013) mRNA expression in the DPBS group (Figure 2F).

In vivo magnetic resonance imaging (MRI) showed that older DPBS animals exhibited a higher prevalence of IFP‐synovitis and bone marrow lesions compared to younger animals, as assessed using a modified MOAKS scoring system previously published [27], indicating a progression toward osteoarthritic changes with age (Figure 2G,H). These findings were consistent with previous studies showing inflammatory and catabolic changes in aged DH guinea pigs, thus supporting the use of this model for evaluating disease‐modifying therapies in primary OA [25].

To further define the disease stage at treatment initiation, we additionally examined knee joints from an independent exploratory cohort of 6‐month‐old DH guinea pigs. TB staining and OARSI scoring revealed mild but detectable OA‐related cartilage alterations at this age, including early surface irregularity and reduced proteoglycan staining, consistent with early‐stage spontaneous OA in this model (Figure S1) [26].

### 2.2 Flow Cytometric Characterization of CD31^+^ SVF Cell Population

**FIGURE 3:**
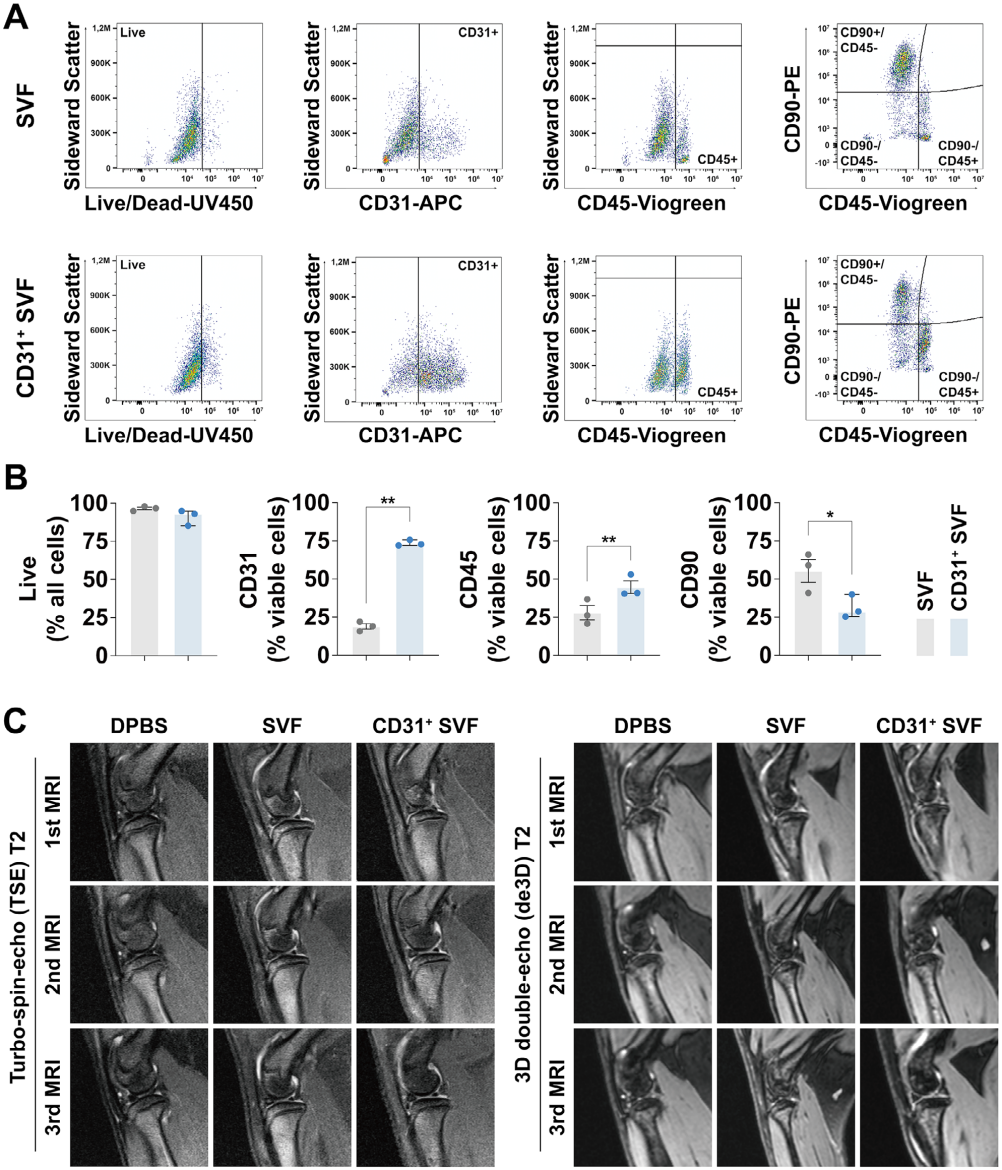
Cell sorting characterization and safety evaluation of IA SVF and CD31^+^ SVF cell administration in the DH guinea pig model. (A and B) Flow cytometric analysis of the cellular composition of SVF and CD31^+^ SVF before and after sorting. (C) MRI‐based safety evaluation following IA injections of SVF and CD31^+^ SVF, with focus on IFP synovitis, ectopic bone formation, osteochondral defects, and joint misalignment. Data points are presented as means ± SEM (B). Statistical comparisons were performed using the Paired Sample T‐Test. ^*^ *p* < 0.05, and ^**^ *p* < 0.01.

To confirm live/dead cell ratios and CD31^+^ cell enrichment before and after sorting, flow cytometric analysis was performed. The CD31^+^ fraction exhibited a similar live cell percentage (96.5 ± 0.9%) as the input SVF (91.1 ± 2.9%, *p* = 0.139). The CD31^+^ fraction contained a significantly higher percentage of CD31^+^ cells (73.4 ± 1.1%) than the input SVF (18.9 ± 1.8%, *p* = 0.002), confirming the successful isolation of CD31^+^ cells through sorting. Additionally, after CD31 sorting, the CD31^+^ fraction had a significantly higher proportion of CD45^+^ cells and a lower proportion of CD90^+^ cells than the input SVF (CD45: 44.8 ± 4.1% vs. 27.9 ± 4.7%, *p* = 0.008; CD90: 31.3 ± 4.4% vs. 55.3 ± 7.5%, *p* = 0.032) (Figure 3A,B). These results demonstrate successful CD31^+^ cell enrichment and subsequent changes in cell composition, potentially impacting the therapeutic behavior of the sorted cell fraction.

### 2.3 MRI‐based Safety Assessment of IA SVF and CD31^+^ SVF Cell Injections

Following model validation, thirty 6‐month‐old guinea pigs (15 males and 15 females) were randomly assigned to receive a single IA injection of DPBS, SVF, or CD31^+^ SVF (*n* = 10 per group, 5 males and 5 females) (Figure 1A). Animals were monitored for 6 months post‐injection for safety and therapeutic outcomes (Figure 1B). All injections were performed under general isoflurane anesthesia, with post‐procedure analgesia and regular health monitoring. MRI‐based morphometry revealed no acute (1 month) or delayed (6 months) adverse reactions following IA injections of SVF or CD31^+^ SVF, with no signs of local immune reactions. Weekly monitoring of body weight, body temperature, injection site appearance, and animal behavior showed no pathological changes, and all guinea pigs maintained stable vital signs throughout the study. Additionally, MRI data demonstrated no radiological evidence of disruption of joint integrity related to cell administrations. Specifically, no abnormal increase in IFP synovitis was observed in any treated animals. Moreover, there was no intra‐ or extra‐articular ectopic bone formation, full‐thickness osteochondral defects, or misalignment of joint structures (Figure 3C). These findings confirm that IA injections of SVF and CD31^+^ SVF were well tolerated and did not induce detectable structural abnormalities or inflammatory reactions, supporting their safety in the DH guinea pig model.

### 2.4 Treatment With SVF and CD31^+^ SVF Mitigates OA Pathology With CD31^+^ SVF Being More Effective

**FIGURE 4:**
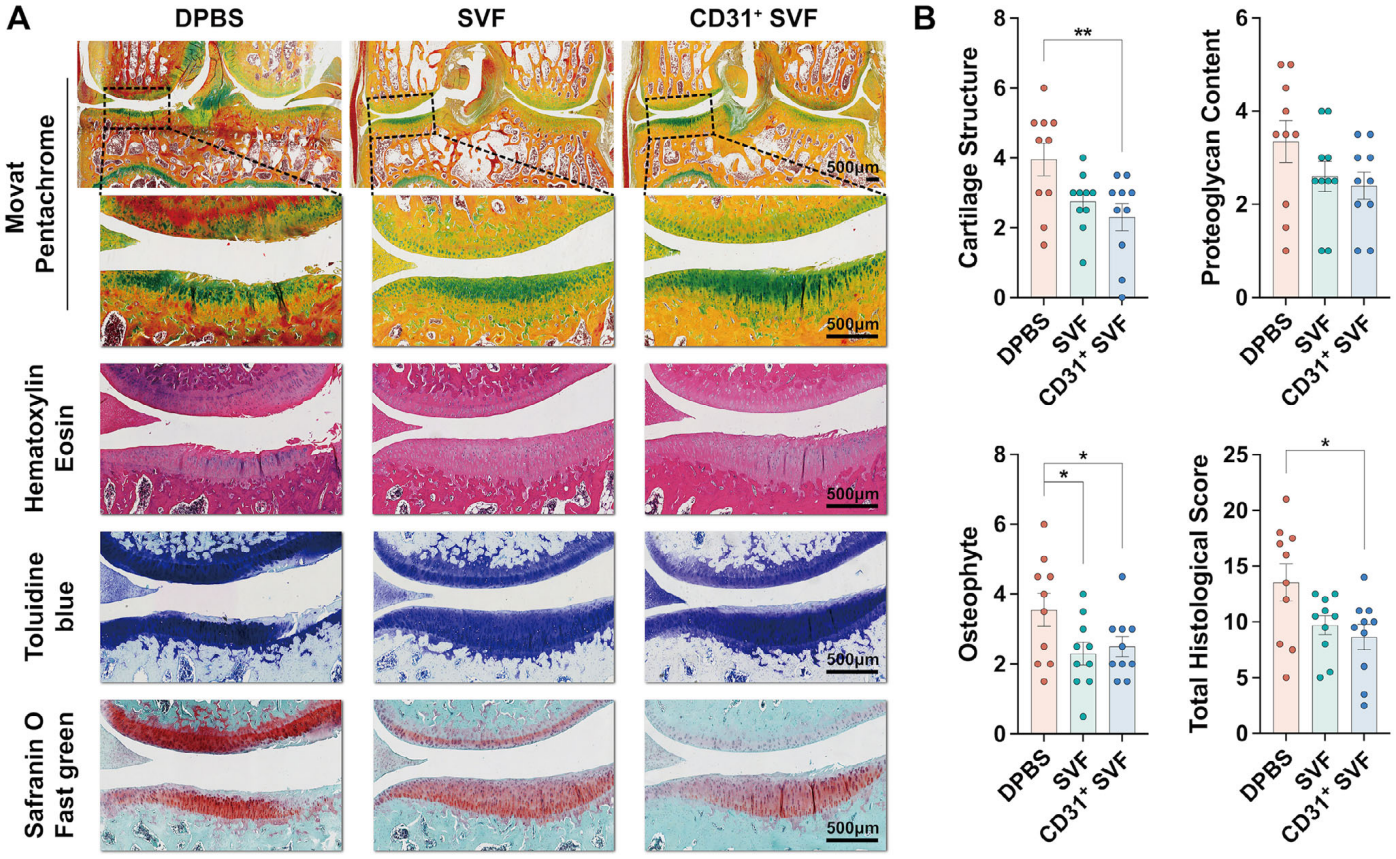
Treatment with SVF and CD31^+^ SVF mitigates OA pathology with CD31^+^ SVF showing broader trends of improvement. (A) Representative histological images of medial knee joint sections stained with Movat's pentachrome, H&E, SO‐FG, and TB to evaluate cartilage degeneration across groups. (B) Histopathological OARSI scores across multiple parameters, including cartilage structure, proteoglycan content, and osteophyte formation. Individual data points are presented as means ± SEM. Statistical comparisons were conducted using two‐way ANOVA with Tukey's post hoc test. ^*^ *p* < 0.05 and ^**^ *p* < 0.01.

To evaluate overall therapeutic effects of SVF and CD31^+^ SVF during OA progression, we conducted histological assessments using Movat's pentachrome, H&E, Safranin‐O and Fast Green (SO‐FG), and TB staining to determine the degree of OA‐related cartilage degeneration (Figure 4A). OARSI scoring of knee joints indicated that all groups exhibited moderate to severe OA‐related alterations in the load‐bearing medial tibial cartilage. However, SVF and CD31^+^ SVF treatment groups tended to exhibit lower histopathological OARSI scores across multiple parameters, including cartilage structure, proteoglycan content, and osteophyte formation, compared to the DPBS group. In the SVF group, a significant improvement was observed in osteophyte formation (*p* = 0.014), while cartilage structure (*p* = 0.066) and total histological scores (*p* = 0.062) showed a trend toward significance. The group means for total histological score were 13.55 ± 1.67 in the DPBS group (range, 5.00–21.00; 95% CI, 9.77–17.33), 9.70 ± 0.86 in the SVF group (range, 5.00–12.50; 95% CI, 7.75–11.65), and 8.65 ± 1.13 in the CD31^+^ SVF group (range, 2.50–14.00; 95% CI, 6.10–11.21). Notably, the CD31^+^ SVF group demonstrated statistically significant improvements across three parameters, including cartilage structure (*p* = 0.009), osteophyte formation (*p* = 0.043), and total histological score (*p* = 0.015). No significant differences were observed between the SVF and CD31^+^ SVF groups (Figure 4B).

### 2.5 SVF and CD31^+^ SVF Alleviate ECM Dysregulation in OA Cartilage, With CD31^+^ SVF Showing Enhanced Remodeling Effects

The homeostasis of cartilage relies on a balanced ECM turnover, ensuring both structural integrity and biomechanical function [28]. To investigate the molecular basis underlying cartilage‐protective effects, we employed matrix‐assisted laser desorption/ionization mass spectrometry imaging (MALDI‐MSI) to assess treatment‐induced changes in ECM protein composition and spatial distribution in the superficial cartilage [29].

In total, MALDI‐MSI analysis of the investigated tissue regions yielded 1395 mass spectra (DPBS: 422; SVF: 454; CD31^+^ SVF: 519), with 606 aligned *m/z* features detected within the tryptic peptide range of *m/z* 600–3200 (Table S1). Of these, 126, 307, and 305 *m/z* values demonstrated discriminatory potential in the comparisons of DPBS vs. SVF, DPBS vs. CD31^+^ SVF, and SVF vs. CD31^+^ SVF, respectively, as determined by receiver operating characteristic (ROC) curve analysis [area under the curve (AUC) < 0.4 or > 0.6] and Wilcoxon rank‐sum test (*p* < 0.05). Integration of MALDI‐MSI and nano‐LC‐ESI‐MS/MS data enabled the preliminary identification of 501 discriminative *m/z* features across all groups, as shown in Table S2. Subsequent validation identified 64 proteins supported by at least two corresponding peptides shown in Table S3. Among these, consistent signal intensity distributions were confirmed for 10 peptides corresponding to five proteins, as detailed in Table S4.

**FIGURE 5:**
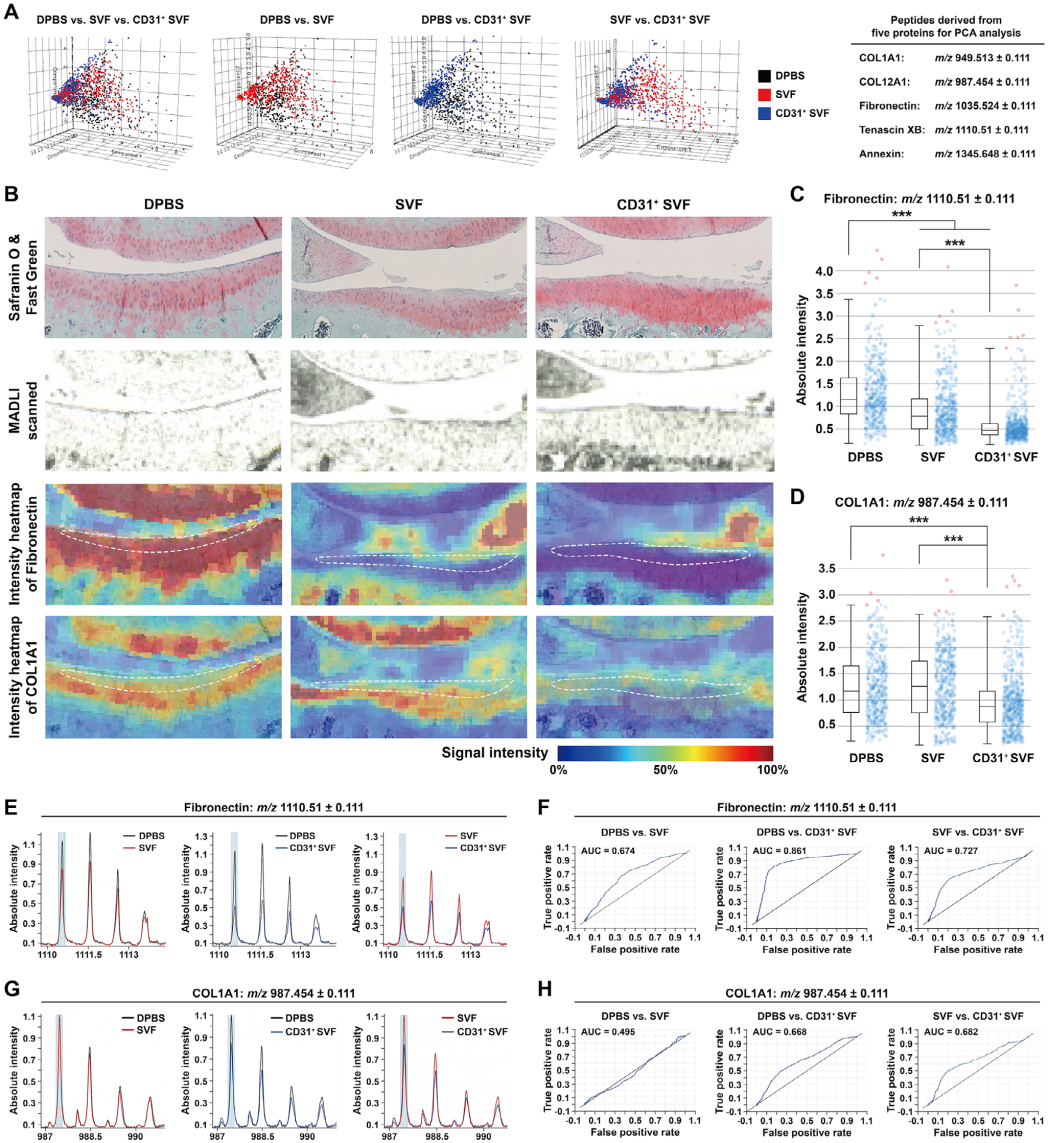
Spatial proteomic analysis using MALDI‐MSI shows that SVF and CD31^+^ SVF treatments alleviate ECM dysregulation in OA cartilage with distinct proteomic remodeling profiles. (A) PCA analysis of peptides derived from five proteins identified across DPBS, SVF, and CD31^+^ SVF groups, illustrating group‐specific clustering and separation of peptide profiles. (B) Representative histological images of SO‐FG staining, grayscale MALDI‐MSI scans, and corresponding intensity heatmaps for fibronectin and COL1A1 peptide distribution across treatment groups. (C and D) Relative quantification of peptide signal intensity for fibronectin (C) and COL1A1 (D) in the superficial cartilage. (E and G) MALDI‐MSI spectra displaying the identified peptides (m/z values) of fibronectin (E) and COL1A1 (G) in the superficial cartilage of indicated groups. (F and H) ROC curve analyses evaluating discrimination capacity of identified fibronectin (F) and COL1A1 (H) peptide peaks between groups. White dashed lines outline the ROIs for analysis. Wilcoxon rank‐sum test analyses were performed to identify discriminative MALDI‐MSI m/z values. ^***^ *p* < 0.001.

To visualize treatment‐induced molecular alterations, principal component analysis (PCA) of the peptides derived from the five identified protein signatures revealed distinct clustering among the DPBS, SVF, and CD31^+^ SVF groups. DPBS samples formed a separate cluster, while SVF and CD31^+^ SVF groups exhibited greater similarity but only partial overlap in their peptide intensity profiles (Figure 5A). This indicates that although both treatments modulate ECM composition, they likely engage distinct and partly independent molecular remodeling mechanisms.

Among the identified proteins, fibronectin, a glycoprotein that facilitates ECM remodeling and participates in pro‐inflammatory signaling, exhibited significantly higher intensity in the DPBS group compared to the SVF and CD31^+^ SVF groups (Figure 5B,C,E,F). Increased fibronectin expression is a characteristic feature of OA pathology, contributing to chondrocyte‐mediated ECM turnover and cartilage degradation [30, 31]. alpha‐1 chain of type I collagen (COL1A1), typically found in fibrocartilage rather than hyaline cartilage [32, 33], showed higher signal intensity in the DPBS group than in the CD31^+^ SVF group, aligning with its role in cartilage fibrosis and ECM disorganization in OA [32] (Figure 5B,D,G,H). Similarly, alpha‐1 chain of type XII collagen (COL12A1), another collagen protein involved in ECM organization, showed significantly higher intensity in the DPBS group than in the SVF and CD31^+^ SVF groups (Table S4). Tenascin XB, an ECM glycoprotein that provides structural support and modulates cell‐matrix interactions [34], was detected at lower levels in the DPBS group, while SVF and CD31^+^ SVF treatments led to an increased intensity (Table S4). This suggests that both treatments may contribute to improved matrix organization and structural integrity of cartilage. In contrast to ECM‐related proteins, annexin, a membrane‐binding protein involved in inflammatory regulation and apoptosis, has been reported to be increased in OA cartilage and associated with chondrocyte stress responses and catabolic signaling [35, 36]. Consistent with this, our data showed higher annexin intensity in the DPBS group compared to the CD31^+^ SVF group, suggesting that cell treatment may mitigate inflammation‐associated annexin expression (Table S4).

Compared to SVF, CD31^+^ SVF‐treated cartilage exhibited lower intensities of fibronectin, COL1A1, COL12A1, and annexin, indicating its ability to steer ECM remodeling and cellular responses to inflammation in OA. In contrast, the relatively lower tenascin XB intensity in CD31^+^ SVF‐treated tissues compared to SVF may indicate a distinct pattern of ECM remodeling or structural reorganization. These data suggest that while both cell treatments attenuated ECM dysregulation in OA cartilage and suppressed cartilage degradation, CD31^+^ SVF may provide superior cartilage protection through distinct molecular pathways.

### 2.6 SVF and CD31^+^ SVF Exert Comparable Protection Against Cartilage Degradation and Fibrotic Remodeling via Partially Different Molecular Mechanisms

**FIGURE 6:**
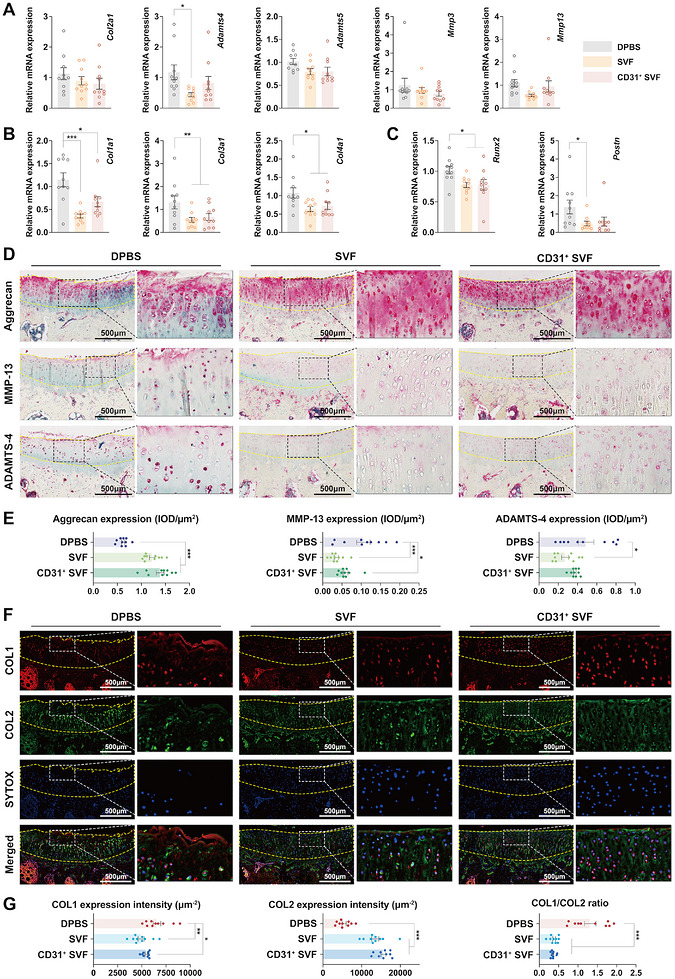
IA administration of SVF and CD31^+^ SVF preserves cartilage integrity and mitigates OA‐associated catabolic and fibrotic activity. (A–C) RT‐qPCR analysis of anabolic and catabolic (A), fibrotic (B), and OA‐associated genes (C) in medial tibial cartilage across groups. (D and E) Immunohistochemical staining and quantification (IOD) of aggrecan, ADAMTS‐4, and MMP‐13 in the medial tibial cartilage. (F and G) Immunofluorescence staining and fluorescence intensity quantification of COL1 and COL2 in the medial tibial cartilage (F). Fluorescence intensity ratio of COL1 to COL2 (G). Yellow dashed lines outline the ROIs for analysis; magnified views are shown for each group. Individual data points are presented as means ± SEM (A–C, E, and G). Statistical comparisons were conducted using two‐way ANOVA with Tukey's post hoc test. ^*^ *p* < 0.05, ^**^ *p* < 0.01, and ^***^ *p* < 0.001.

Given the cartilage‐protective effects observed in histological and spatial proteomic analyses, we next explored key mRNA expression profiles in medial tibial cartilage using real‐time quantitative polymerase chain reaction (RT‐qPCR), focusing on cartilage anabolic, catabolic, and fibrotic markers. The expression of the anabolic marker *Col2a1* showed no significant differences among DPBS, SVF, and CD31^+^ SVF groups. However, among catabolic genes, *Adamts4* showed reduced expression levels following treatment with SVF (*p* = 0.011). In the CD31^+^ SVF group, *Adamts4* expression levels also tended to be lower relative to DPBS, though without reaching statistical significance. Similarly, *Adamts5*, *Mmp3*, and *Mmp13* mRNA levels showed a modest decrease in both cell‐treated groups, but the differences did not meet significance thresholds (Figure 6A). Fibrotic genes showed markedly decreased expression following SVF and CD31^+^ SVF treatment. Expression levels of *Col1a1*, alpha‐1 chain of type III collagen (*Col3a1*), and alpha‐1 chain of type IV collagen (*Col4a1*) were significantly reduced in both treatment groups compared to DPBS (*Col1a1*: *p* < 0.001 vs. SVF, *p* = 0.015 vs. CD31^+^ SVF; *Col3a1*: *p* = 0.002 vs. SVF, *p* = 0.009 vs. CD31^+^ SVF; *Col4a1*: *p* = 0.012 vs. SVF, *p* = 0.049 vs. CD31^+^ SVF) (Figure 6B). Similarly, OA‐associated markers runt‐related transcription factor 2 (*Runx2*) and periostin (*Postn*), which contribute to cartilage degeneration and osteophyte formation, were significantly reduced following treatment with SVF and CD31^+^ SVF. *Runx2* expression was significantly lower in both SVF (*p* = 0.040) and CD31^+^ SVF (*p* = 0.044) groups compared to DPBS, while *Postn* expression was significantly reduced in the SVF group (*p* = 0.032) (Figure 6C). No significant differences were observed between SVF and CD31^+^ SVF groups, further suggesting that both cell types exert similar cartilage‐protective effects.

Given the limited transcriptional differences in cartilage remodeling‐related markers, we examined protein‐level changes of key anabolic and catabolic cartilage markers via immunohistochemistry to explore the apparent cartilage‐protective effects observed histologically. Aggrecan, a relevant component of cartilage ECM, was significantly elevated following treatment with SVF and CD31^+^ SVF, as indicated by a higher IOD compared to the DPBS group (*p* < 0.001 for both SVF vs. DPBS and CD31^+^ SVF vs. DPBS). The mean aggrecan IOD values were 0.62 ± 0.03 in the DPBS group (range, 0.45–0.81; 95% CI, 0.54–0.69), 1.22 ± 0.06 in the SVF group (range, 1.02–1.53; 95% CI, 1.10–1.35), and 1.39 ± 0.08 in the CD31^+^ SVF group (range, 0.92–1.71; 95% CI, 1.21–1.57). Correspondingly, catabolic activity contributing to ECM degradation was attenuated in both treatment groups. For metalloproteinase (MMP)‐13, a significant decrease in IOD was observed in both SVF (*p* < 0.001) and CD31^+^ SVF groups (*p* = 0.013) compared to DPBS (Figure 6D,E). The mean MMP‐13 IOD values were 0.11 ± 0.02 in the DPBS group (range, 0.03–0.19; 95% CI, 0.07–0.15), 0.04 ± 0.01 in the SVF group (range, 0.01–0.07; 95% CI, 0.02–0.05), and 0.06 ± 0.01 in the CD31^+^ SVF group (range, 0.04–0.11; 95% CI, 0.04–0.07). ADAMTS‐4, which mediates aggrecan breakdown, was significantly decreased only in the SVF group, as shown by IOD (*p* = 0.012 vs. DPBS). These findings are consistent with histological evidence of cartilage preservation following cell therapy.

To validate the impact of SVF and CD31^+^ SVF on cartilage remodeling based on spatial proteomics results, we investigated cartilage homeostasis by evaluating the protein expression levels of COL1, a fibrosis marker, and COL2, an anabolic cartilage marker, in cartilage tissue. Our findings revealed significantly higher COL1 expression and lower COL2 expression in the DPBS group compared to both SVF and CD31^+^ SVF groups (COL1: *p* = 0.002 vs. SVF, *p* = 0.039 vs. CD31^+^ SVF; COL2: *p* < 0.001 vs. both SVF and CD31^+^ SVF). Notably, the COL1/COL2 expression ratio was significantly decreased in both treatment groups compared to the DPBS group (*p* < 0.001 for both SVF and CD31^+^ SVF), suggesting attenuation of cartilage fibrosis and preservation of native matrix integrity (Figure 6F,G). No significant differences were observed between the SVF and CD31^+^ SVF groups in terms of COL1, COL2, or COL1/COL2 ratio.

To further validate tissue remodeling‐related changes at the protein level, we stained slides for POSTN and RANKL. POSTN expression in medial tibial cartilage and periosteal tissue was markedly elevated in the DPBS group, particularly in cartilage and peri‐osteophytic regions, whereas both SVF and CD31^+^ SVF treatments reduced POSTN protein expression relative to DPBS, and the reduction was significantly greater in the CD31^+^ SVF group than in the SVF group (Figure S2A). In addition, RANKL expression in medial tibial cartilage was elevated in the DPBS group and reduced in both treatment groups (Figure S2B), underlining attenuation of OA‐associated remodeling‐related signaling. Together, these findings are aligned with RT‐qPCR data. Attenuation of ECM disorganization, osteophyte‐associated remodeling, and cartilage–bone interface‐related signaling were seen following both treatments.

**FIGURE 7:**
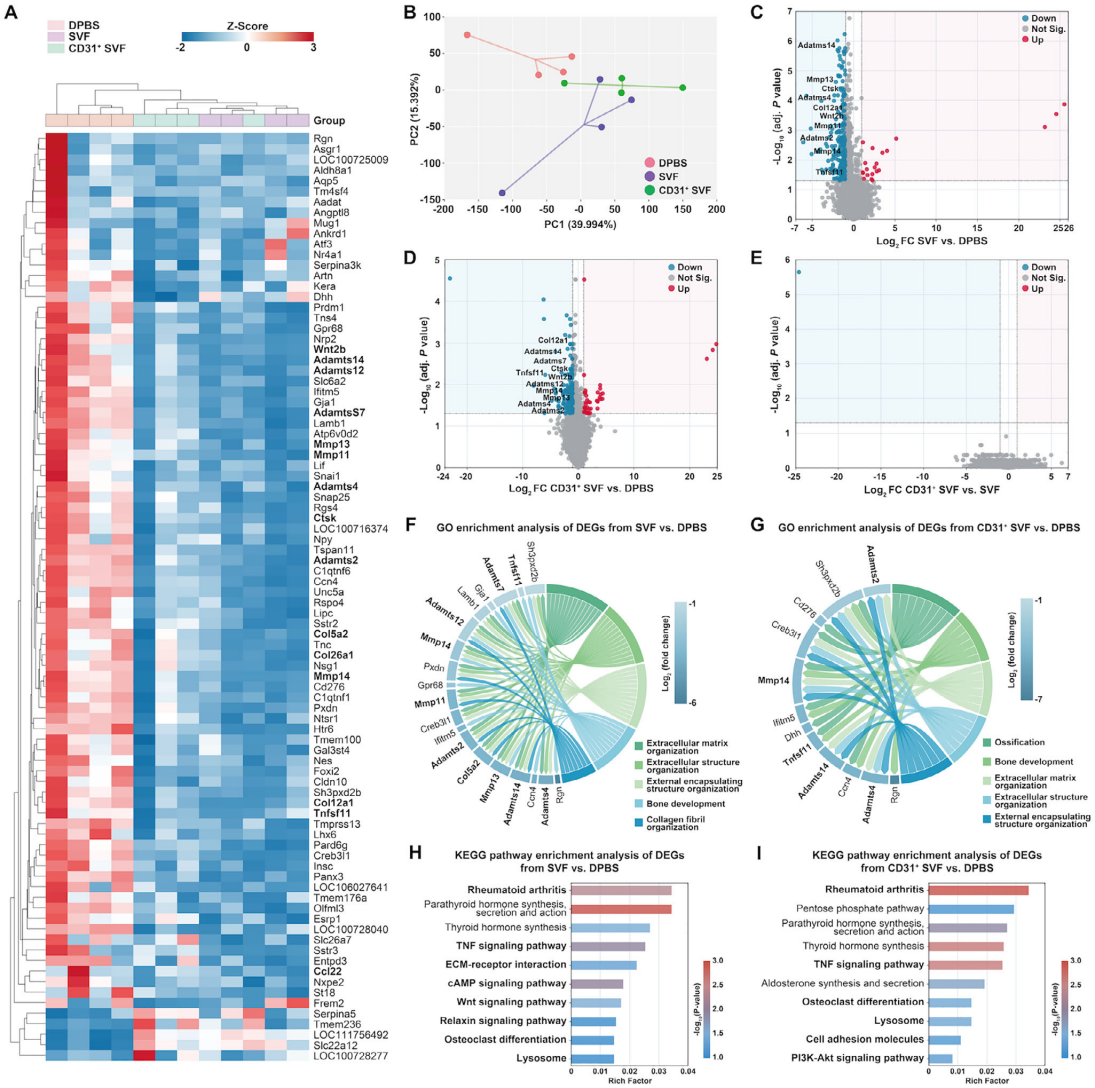
Transcriptomic analyses reveal comparable cartilage‐protective effects of SVF and CD31^+^ SVF treatments via partially distinct molecular pathways. (A) Hierarchical clustering heatmap of DEGs across DPBS, SVF, and CD31^+^ SVF groups (*n* = 4 per group). (B) PCA of global gene expression profiles in medial tibial cartilage. (C–E) Volcano plots showing DEGs identified in pairwise comparisons: SVF vs. DPBS (C), CD31^+^ SVF vs. DPBS (D), and CD31^+^ SVF vs. SVF (E) (criteria: adj. *P* < 0.05, |log_2_FC| > 1). (F,G) GO enrichment analysis of DEGs from SVF vs. DPBS (F) and CD31^+^ SVF vs. DPBS (G) comparisons. (H,I) KEGG pathway enrichment analysis of DEGs from SVF vs. DPBS (H) and CD31^+^ SVF vs. DPBS (I) comparisons.

To investigate the molecular mechanisms underlying the observed protein‐level cartilage protection by SVF and CD31^+^ SVF treatments, we conducted a comprehensive transcriptomic analysis of medial tibial cartilage. Hierarchical clustering of differentially expressed genes (DEGs) (excluding unannotated genes), applying adj. *P* < 0.05 and absolute log_2_ fold change (|log_2_FC|) > 1 as criteria, showed distinct expression profiles across the three groups. Samples from each group clustered tightly, except for one CD31^+^ SVF sample that grouped with SVF (Figure 7A). PCA further confirmed that global gene expression patterns in the SVF and CD31^+^ SVF groups were clearly distinct from the DPBS control, while the two groups only partially overlapped (Figure 7B). Next, we performed pairwise comparisons and found that the SVF vs. DPBS comparison yielded 238 DEGs, with 217 downregulated genes, including key OA‐related markers such as *Adamts4* and *Mmp13*, and 21 upregulated genes (Figure 7C). The CD31^+^ SVF vs. DPBS comparison identified 210 DEGs, including 172 downregulated genes such as *Adamts4* and *Mmp13*, and 38 upregulated genes (Figure 7D). Notably, the CD31^+^ SVF vs. SVF comparison yielded only one DEG, demonstrating the similarity of cartilage‐modulating effects between both treatments (Figure 7E). Gene ontology (GO) enrichment analysis of SVF vs. DPBS DEGs showed enriched biological processes related to ECM‐remodeling, bone development, and collagen fibril organization, all relevant to OA pathophysiology (Figure 7F). Similarly, GO analysis of CD31^+^ SVF vs. DPBS DEGs showed enrichment in ECM‐remodelling, bone development, and ossification (Figure 7G). Complementary Kyoto Encyclopedia of Genes and Genomes (KEGG) pathway analysis showed that SVF vs. DPBS DEGs were significantly enriched in rheumatoid arthritis‐associated, TNF, Wnt, and cAMP signaling pathways (Figure 7H), while CD31^+^ SVF vs. DPBS DEGs were enriched in rheumatoid arthritis‐associated, TNF, and PI3K‐Akt signaling pathways (Figure 7I).

Taken together, these findings demonstrate that both SVF and CD31^+^ SVF treatments mitigate cartilage degradation and fibrosis by reducing catabolic and fibrotic gene expression, albeit through partially distinct upstream signaling pathways.

### 2.7 SVF and CD31^+^ SVF Cell Treatments Attenuate Joint Inflammation via Partially Distinct Transcriptional Responses but Do Not Alter Systemic Inflammation

**FIGURE 8:**
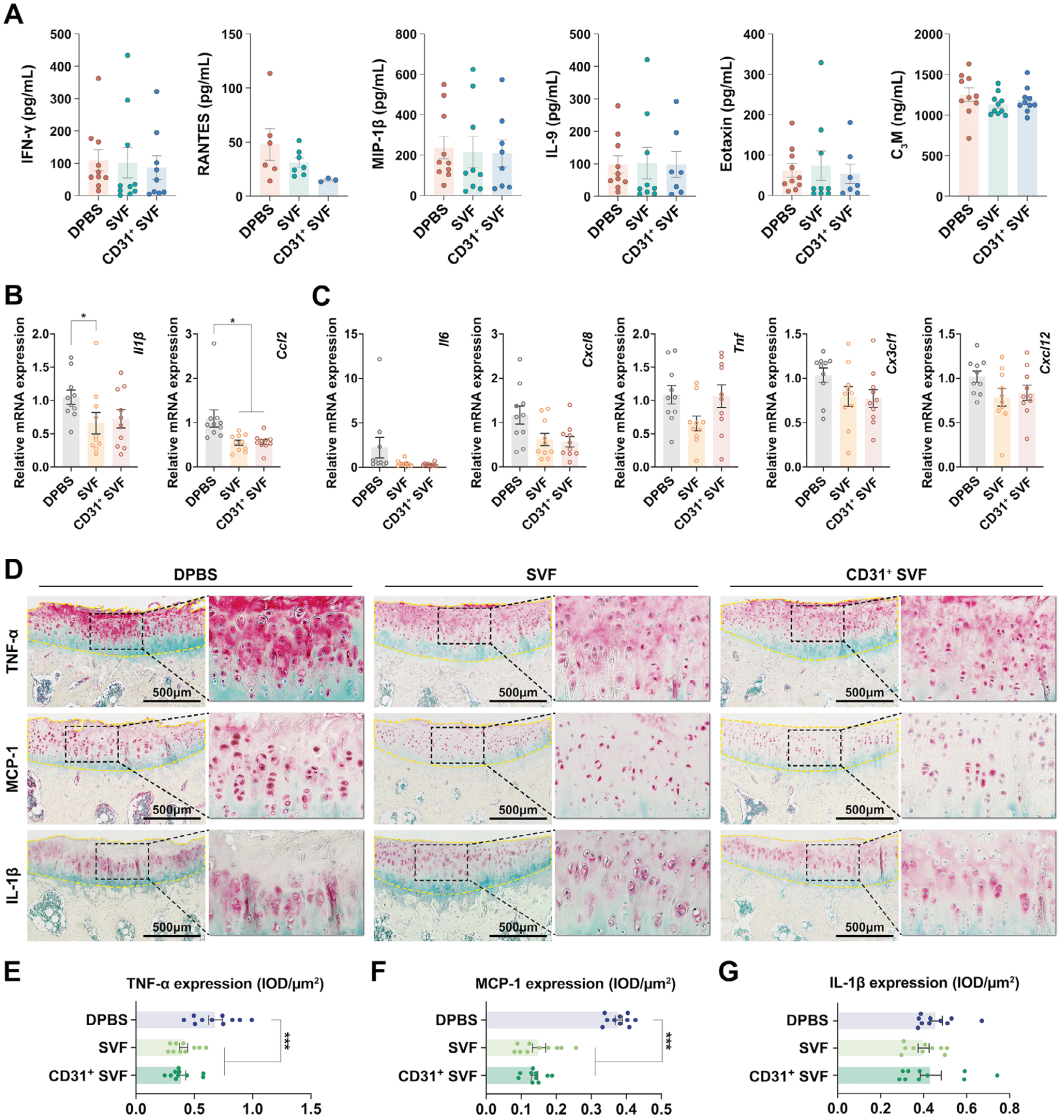
IA SVF and CD31^+^ SVF treatments do not influence systemic inflammatory markers, but reduce local inflammatory activity in cartilage. (A) Serum ELISA analysis of inflammatory markers, including IFN‐γ, RANTES, MIP‐1β, IL‐9, Eotaxin, and C_3_M, showing no significant differences between groups. (B and C) Quantitative PCR analysis of pro‐inflammatory cytokines, including *Il1β* and *Ccl2* (B), as well as *Il6*, *Cxcl8, Tnf, Cx3cl1*, and *Cxcl12* (C) in the IFP. (D) Immunohistochemical staining of TNF‐α, MCP‐1, and IL‐1β in the medial tibial cartilage. (E–G) Quantification (IOD) of TNF‐α (E), MCP‐1 (F), and IL‐1β (G). Yellow dashed lines in (D) outline the ROIs for analysis; magnified views are shown for each group. Individual data points are presented as means ± SEM (A–C and E–G). Statistical comparisons were performed using two‐way ANOVA with Tukey's post hoc test. ^*^ *p* < 0.05, ^**^ *p* < 0.01, and ^***^ *p* < 0.001.

Given the critical role of inflammation in driving cartilage degradation and OA progression, we investigated whether SVF and CD31^+^ SVF treatments attenuate inflammatory responses both systemically and within joint tissues. We first assessed systemic inflammation beyond the local joint environment by measuring serum levels of inflammatory cytokines. A multiplex immunoassay targeting inflammatory cytokines, including interferon‐gamma (IFN‐γ), regulated upon activation normal T expressed and secreted (RANTES), macrophage inflammatory protein‐1 beta (MIP‐1β), IL‐9, eotaxin, and C_3_M (a degradation product of type III collagen) showed no significant differences in serum levels between the DPBS, SVF, and CD31^+^ SVF groups (Figure 8A), suggesting that IA cell delivery does not influence systemic inflammatory responses.

We next assessed local anti‐inflammatory effects of SVF and CD31^+^ SVF treatments on joint tissues by investigating mRNA expression of pro‐inflammatory cytokines in the IFP, a major contributor to OA‐related inflammation. The mRNA expression of *Il1β* and *Ccl2* was lower in the SVF and CD31^+^ SVF treatment groups than in the DPBS group, with statistically significant reductions observed for *Il1β* (*p* = 0.024 for SVF vs. DPBS) and *Ccl2* (*p* = 0.036 for SVF vs. DPBS; *p* = 0.044 for CD31^+^ SVF vs. DPBS) (Figure 8B). Additionally, other OA‐associated inflammatory cytokines, including *Il6*, chemokine C‐X‐C motif ligand (*Cxcl*) *8*, *Tnf*, C‐X3‐C motif chemokine ligand 1 (*Cx3cl1*), and *Cxcl12* exhibited a trend toward lower expression in both the SVF and CD31^+^ SVF groups compared to DPBS, although not reaching statistical significance (Figure 8C). No significant differences were detected between SVF and CD31^+^ SVF treatment groups.

To determine whether these transcriptional observations translated to protein‐level changes, we next evaluated the expression of key inflammatory mediators in medial tibial cartilage using immunohistochemistry. Quantification of TNF‐α expression in medial tibial cartilage revealed a significant reduction in averaged IOD in groups treated with SVF or CD31^+^ SVF compared to DPBS controls (*p* < 0.001 for both SVF vs. DPBS and CD31^+^ SVF vs. DPBS) (Figure 8D,E). The mean TNF‐α IOD values were 0.68 ± 0.06 in the DPBS group (range, 0.41–0.99; 95% CI, 0.55–0.82), 0.41 ± 0.03 in the SVF group (range, 0.28–0.60; 95% CI, 0.33–0.49), and 0.39 ± 0.35 in the CD31^+^ SVF group (range, 0.25–0.58; 95% CI, 0.31–0.47). Similarly, the averaged IOD of MCP‐1 was significantly lower in both treatment groups than in the DPBS group (*p* < 0.001 for both SVF vs. DPBS and CD31^+^ SVF vs. DPBS) (Figure 8D,F). No significant differences were detected between the two treatment groups for either marker. Although quantification of IL‐1β expression (averaged IOD) in medial tibial cartilage did not show significant differences between groups, the DPBS group exhibited pronounced histological abnormalities, including chondrocyte clustering, hypercellularity, and regions of diffuse hypocellularity (Figure 8D,G). These findings suggest that both SVF and CD31^+^ SVF attenuate local inflammatory activity within the knee joint, which may contribute to the observed cartilage preservation.

**FIGURE 9:**
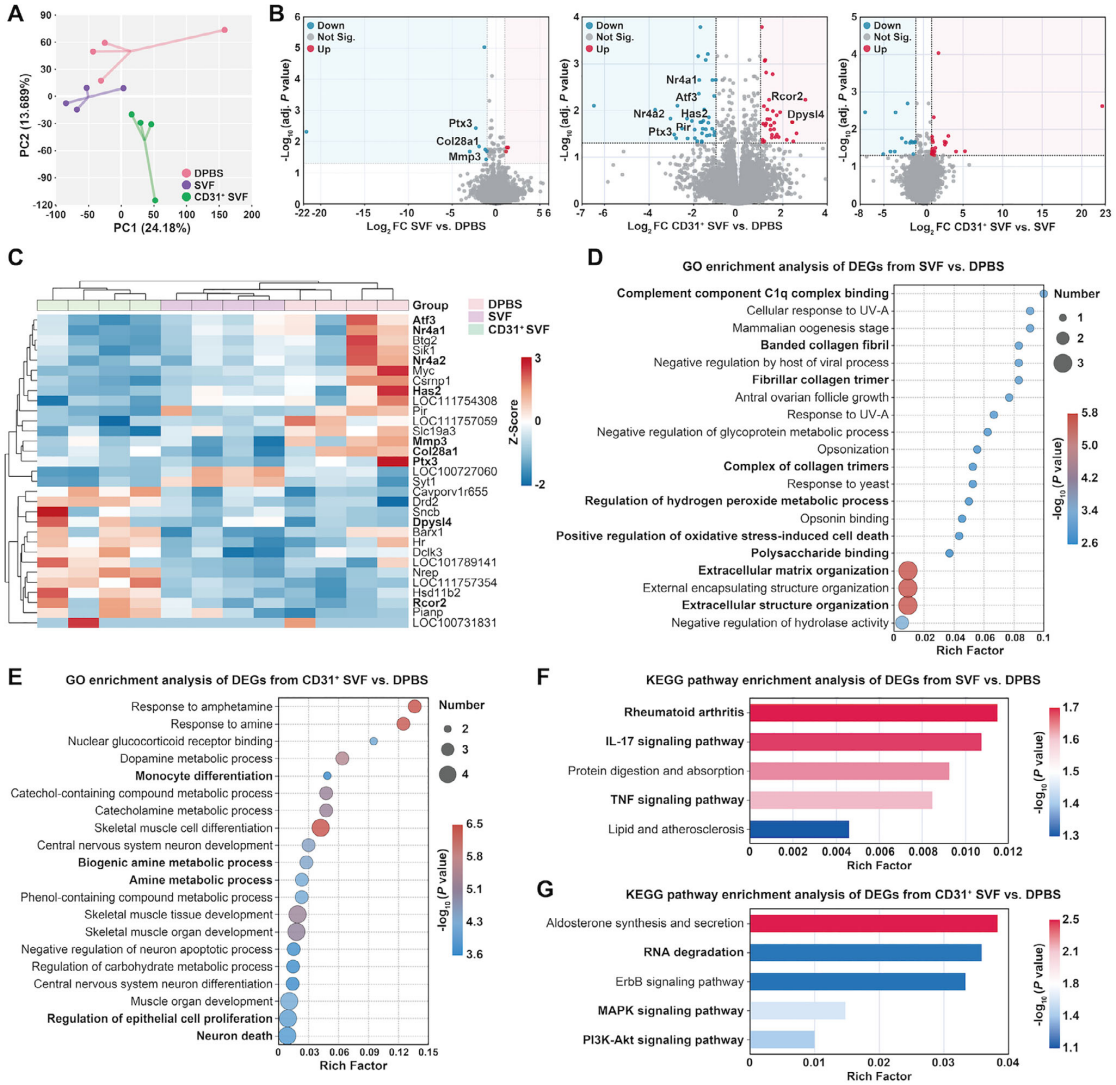
Transcriptomic analyses reveal anti‐inflammatory effects of SVF and CD31^+^ SVF treatments in the IFP via partially distinct transcriptional responses. (A) PCA of global gene expression profiles in IFP samples from DPBS, SVF, and CD31^+^ SVF groups (*n* = 4 per group). (B) Volcano plots showing DEGs identified in pairwise comparisons: SVF vs. DPBS, CD31^+^ SVF vs. DPBS, and CD31^+^ SVF vs. SVF (criteria: adj. *P* < 0.05, |log_2_FC| > 1). (C) Hierarchical clustering heatmap of DEGs. (D and E) GO enrichment analysis of DEGs from SVF vs. DPBS (D) and CD31^+^ SVF vs. DPBS (E) comparisons. (F and G) KEGG pathway enrichment analysis of DEGs from SVF vs. DPBS (F) and CD31^+^ SVF vs. DPBS (G) comparisons.

To further elucidate the molecular mechanisms underlying the anti‐inflammatory effects of SVF and CD31^+^ SVF treatments, we conducted a comprehensive transcriptomic analysis of IFP tissues. PCA of global gene expression profiles revealed that both SVF and CD31^+^ SVF groups were clearly distinct from the DPBS control. There was further distinct clustering between the treatment groups, suggesting different transcriptional responses to each intervention (Figure 9A). Next, we performed pairwise comparisons to identify DEGs using the criteria of adjusted *p* value (adj. *P*) < 0.05 and |log_2_FC| > 1. The SVF vs. DPBS comparison yielded 11 DEGs, with SVF downregulating 8 genes, including *Ptx3*, *Col28a1*, and *Mmp3*, and upregulating 3 genes. The CD31^+^ SVF vs. DPBS comparison identified a larger set of 72 DEGs, comprising 38 downregulated genes, including *Ptx3*, *Nr4a1*, and *Nr4a2*, and 34 upregulated genes, such as *Rcor2* and *Dpysl14*. The direct comparison between CD31^+^ SVF vs. SVF yielded 36 DEGs (14 downregulated and 22 upregulated by CD31+ SVF), though none were directly associated with OA‐related pathways (Figure 9B). Hierarchical clustering of DEGs (excluding unannotated genes) further demonstrated transcriptional distinction across groups, with replicates within each group clustering tightly (Figure 9C). GO enrichment analysis of SVF vs. DPBS DEGs identified enriched biological processes related to ECM organization, collagen binding, and oxidative stress regulation, functionally relevant to OA pathophysiology (Figure 9D). In contrast, GO analysis of CD31^+^ SVF vs. DPBS DEGs were enriched in processes such as monocyte differentiation and amine metabolic processes (Figure 9E), reflecting local immune modulation and metabolic activity following CD31^+^ SVF treatment. Complementary KEGG pathway analysis showed that SVF vs. DPBS DEGs were significantly enriched in signaling pathways such as IL‐17, TNF, and those associated with rheumatoid arthritis (Figure 9F), while CD31^+^ SVF vs. DPBS DEGs were enriched in pathways such as PI3K‐Akt and MAPK signaling pathways (Figure 9G), all of which are implicated in joint inflammation and OA pathogenesis. GO and KEGG analyses of DEGs from the CD31^+^ SVF vs. SVF comparison did not show prominent OA‐related enrichment.

Together, these findings demonstrate that both SVF and CD31^+^ SVF treatments exert comparable local anti‐inflammatory effects in joint tissues, though through partially distinct molecular pathways.

### 2.8 Regulation of Subchondral Bone Remodeling in OA by SVF and CD31+ SVF Therapy, With CD31^+^ SVF Showing Superior Effects on Trabecular Integrity

**FIGURE 10:**
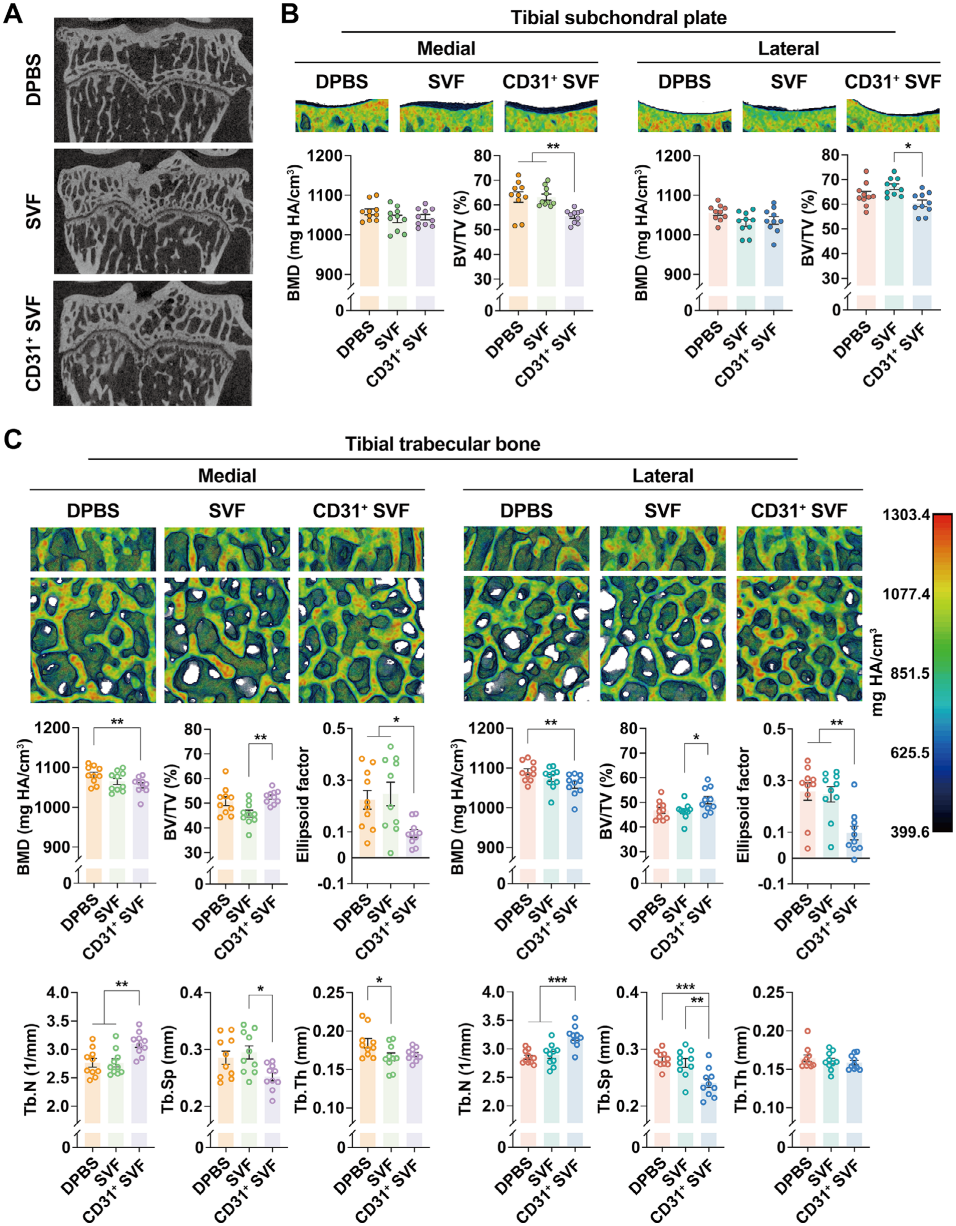
Effects of SVF and CD31^+^ SVF cell treatment on subchondral bone remodeling and trabecular bone microstructure in OA. (A) Representative 2D µCT coronal‐section images of the tibial subchondral plate and trabecular bone. (B) Representative 3D reconstructions of the tibial subchondral plate and quantitative analysis, including BMD and BV/TV, with comparisons between groups. (C) Representative 3D reconstructions of tibial trabecular bone and quantitative analysis, including BMD, BV/TV, ellipsoid factor, Tb.N, Tb.Sp, and Tb.Th, with comparisons between groups. Individual data points are presented as means ± SEM (B and C). Statistical comparisons were performed using two‐way ANOVA with Tukey's post hoc test. ^*^ *p* < 0.05, ^**^ *p* < 0.01, and ^***^ *p* < 0.001.

Alterations in both the subchondral plate and trabecular bone contribute to OA disease progression [37]. Increased subchondral stiffness can negatively impact the overlying articular cartilage by disrupting mechanical load distribution and thus exacerbate cartilage degeneration [38]. Trabecular bone, which serves as the primary structural support for the joint, undergoes pathological remodeling in OA, including increased bone mineral density (BMD), decreased trabecular number (Tb.N), and greater trabecular separation (Tb.Sp), all of which contribute to joint instability and cartilage damage. Therefore, we performed microcomputed tomography (µCT) analysis of the tibial subchondral region to evaluate whether SVF and CD31^+^ SVF therapies would alter pathological bone changes. Representative 2D coronal‐section images are shown in Figure 10A.

In the subchondral plate, the SVF group exhibited a reduction in BMD on the lateral side compared to DPBS, showing a trend toward partial mitigation of sclerotic changes, although this difference did not reach statistical significance (*p* = 0.053 vs. DPBS). Notably, CD31^+^ SVF treatment showed a more effective modulation in preserving subchondral plate microarchitecture, as evidenced by significantly lower bone volume ratio (BV/TV) values relative to both SVF and DPBS groups (medial: *p* = 0.004 vs. both DPBS and SVF; lateral: *p* = 0.003 vs. SVF) (Figure 10B). The mean medial subchondral plate BV/TV values were 63.21 ± 2.13 in the DPBS group (range, 51.58–72.14; 95% CI, 58.40–68.03), 63.22 ± 1.23 in the SVF group (range, 59.42–70.05; 95% CI, 60.44–66.00), and 55.47 ± 0.84 in the CD31^+^ SVF group (range, 50.97–59.40; 95% CI, 53.57–57.37). Given that increased BV/TV in the subchondral plate is typically associated with pathological thickening and mineralization [39], this finding suggests a beneficial effect of CD31^+^ SVF on subchondral remodeling. The magnitude of this effect differed between compartments, being more pronounced in the medial subchondral plate, whereas in the lateral subchondral plate BV/TV was significantly lower relative to SVF but not relative to DPBS. This compartment‐dependent pattern is consistent with disease distribution characteristics of the DH guinea pig model, which exhibits more pronounced cartilage degradation medially [40].

In tibial trabecular bone, the CD31^+^ SVF group showed significantly lower BMD compared to the DPBS group (medial: *p* = 0.002; lateral: *p* = 0.003), confirming counteracted trabecular bone sclerosis by CD31^+^ SVF treatment. The mean medial trabecular BMD values were 1081.51 ± 6.88 in the DPBS group (range, 1047.14–1110.73; 95% CI, 1065.95–1097.08), 1065.06 ± 7.67 in the SVF group (range, 1033.51–1099.49; 95% CI, 1047.71–1082.41), and 1055.08 ± 6.59 in the CD31^+^ SVF group (range, 1007.82–1080.81; 95% CI, 1040.17–1069.98). At the same time, the accompanying increases in BV/TV and Tb.N support improved preservation and organization of the trabecular network. Additionally, BV/TV was significantly higher in the CD31^+^ SVF group than in the SVF group (medial: *p* = 0.008; lateral: *p* = 0.037) and tended to be higher compared with the DPBS group on the lateral site (*p* = 0.057), indicating improved trabecular bone volume preservation and microstructural stability. The ellipsoid factor, a parameter that distinguishes rod‐ from plate‐like trabecular geometry, was significantly lower in the CD31^+^ SVF group than in the DPBS and SVF groups (medial: *p* = 0.038 vs. DPBS, *p* = 0.013 vs. SVF; lateral: *p* = 0.004 vs. DPBS, *p* = 0.007 vs. SVF), suggesting a shift toward native bone structure. Tb.N was markedly increased (medial: *p* = 0.005 vs. DPBS, *p* = 0.004 vs. SVF; lateral: *p* < 0.001 vs. both DPBS and SVF), and Tb.Sp was significantly decreased (medial: *p* = 0.019 vs. SVF; lateral: *p* = 0.001 vs. DPBS, *p* = 0.002 vs. SVF) in the CD31^+^ SVF group compared to both DPBS and SVF groups, further supporting improved trabecular organization. Conversely, trabecular thickness (Tb.Th), which increases in OA‐associated remodeling, showed a decreasing trend in both treatment groups compared to the DPBS group, with a significant difference detected between DPBS and SVF groups (*p* = 0.033), suggesting that Tb.Th may also be regulated by SVF and CD31^+^ SVF (Figure 10C). Together, these findings indicate that while both SVF and CD31^+^ SVF therapies partially attenuated OA‐associated subchondral bone remodeling, CD31^+^ SVF exhibited more pronounced and consistent improvements in trabecular bone integrity, supporting its preserving role in joint microarchitecture.

To further examine mechanisms associated with the differential bone‐protective effects of CD31^+^ SVF, we performed CD31/EMCN co‐staining in the osteochondral region. Double immunofluorescence staining for CD31 and EMCN revealed distinct spatial patterns of CD31^hi^EMCN^hi^‐positive vascular‐associated structures across groups. In the DPBS group, CD31^hi^EMCN^hi^‐positive structures were most frequently detected near the calcified cartilage zone and peri‐osteophytic regions. The SVF group showed a reduced abundance of such structures compared with DPBS, whereas the CD31^+^ SVF group exhibited the lowest abundance in these locations (Figure S2C). This distribution is consistent with attenuation of aberrant vascular‐associated remodeling at the osteochondral interface following cell treatment, with a more pronounced effect in the CD31^+^ SVF group.

## 3 Discussion

This study provides a direct comparison of unfractionated SVF and CD31^+^‐enriched SVF IA treatment in a spontaneous primary KOA model and yields three principal findings. First, both SVF and CD31^+^ SVF attenuated OA‐associated cartilage degeneration, ECM dysregulation, and local inflammation in the DH guinea pig model. Second, although CD31^+^ SVF did not uniformly outperform unfractionated SVF across all endpoints, it showed a more consistent pattern of cartilage‐related histopathological improvement and stronger effects in preserving subchondral plate and trabecular bone microarchitecture. Third, spatial proteomic and transcriptomic profiling indicated that SVF and CD31^+^ SVF were associated with partially distinct molecular profiles, with CD31^+^ SVF showing stronger associations with ECM remodeling and PI3K‐Akt/MAPK‐related signaling. Due to substantial depletion of non‐CD31^+^ cell populations during sorting, CD31^+^ cells may be predominantly responsible for the therapeutic activity of bulk SVF. Collectively, these findings position CD31^+^ enrichment as a mechanism‐guided strategy that may refine the therapeutic profile of SVF‐based therapy.

Conflicting clinical evidence has been published regarding the efficacy of SVF or autologous micro‐fragmented adipose tissue in KOA therapy. Recent studies, including a single‐center retrospective randomized controlled study and a multisite prospective double‐blind randomized placebo‐controlled clinical trial with a small sample size, have reported pain reduction, functional improvement, and symptom relief lasting for up to 12 months or longer [41, 42]. However, other prospective single‐blind randomized controlled trials with bigger sample sizes have shown limited or no therapeutic effect [9, 43]. One possible explanation for these conflicting results is that OA is a highly heterogeneous and multifaceted disease group, likely characterized by multiple molecular endotypes [44, 45, 46]. These comprise biologically distinct subsets defined by specific pathophysiological mechanisms [47], and clinical phenotypes [48, 49, 50, 51], which may markedly influence treatment response. While some patients exhibit an inflammatory‐dominant OA endotype with high cytokine activity, other patients present with primarily low‐tissue turnover or structural destruction OA endotypes [44, 45]. A compelling example underscoring the importance of OA endotypes and patient stratification is a recent analysis showing that oral salmon calcitonin failed to demonstrate overall efficacy in an unstratified OA cohort, but significantly reduced pain in patients with a structural damage endotype [52]. While animal models typically reflect a specific OA pathology, they do not capture the full complexity and heterogeneity observed in human OA [53, 54]. Most preclinical OA studies use models of secondary or post‐traumatic OA, whereas corresponding clinical studies have predominantly targeted patients with primary OA [55]. To better reflect the clinical reality and capture key features of primary OA, we employed the DH guinea pig model, a spontaneous KOA model.

The protective effects of SVF in OA are primarily attributed to its immunomodulatory actions and cartilage preservation [13, 14, 56]. SVF mitigates inflammation via paracrine signaling, modulating immune responses [57, 58], suppressing pro‐inflammatory cytokines such as IL‐1β, TNF‐α, and IL‐6, and enhancing anti‐inflammatory mediators such as IL‐10 and transforming growth factor‐beta [13, 14, 59, 60]. It exerts protective effects on cartilage through the inhibition of catabolic enzymes (e.g., MMP‐13 and ADAMTS‐5) [15, 61] and the promotion of anabolic markers (e.g., COL2 and SOX9) through paracrine signaling [13, 59, 60, 62], contributing to ECM stabilization and chondroprotection. Consistent with these findings, both SVF and CD31^+^ SVF treatments in this study significantly reduced inflammatory mediators in the IFP and cartilage (e.g., TNF‐α, and MCP‐1), and attenuated inflammatory signaling pathways such as TNF and those associated with rheumatoid arthritis. In parallel, both treatments enhanced cartilage matrix integrity by increasing aggrecan expression and suppressing MMP‐13 at the protein level, supporting treatment‐driven cartilage preservative effects. Additionally, both treatments significantly attenuated cartilage fibrosis and alleviated ECM disorganization, as evidenced by decreased levels of fibronectin, COL1A1, and COL12A1 in treated cartilage. These findings are consistent with previous studies demonstrating that fibronectin fragments and aberrant collagen expression contribute to cartilage degradation by promoting catabolic enzyme activation and inflammation [30, 63], processes that can be mitigated by adipose‐derived cell populations [64]. Furthermore, POSTN, a matricellular protein implicated in osteophyte formation and ECM disorganization, showed elevated protein expression in the DPBS group and reduced expression following both SVF and CD31^+^ SVF treatment. RANKL expression in medial tibial cartilage was likewise reduced following both treatments relative to DPBS. Together, these findings support the RT‐qPCR data and are consistent with reduced periosteal and ECM remodeling, as well as diminished osteochondral remodeling‐related signaling, following both SVF and CD31^+^ SVF treatment in OA.

Despite their overall similarity, CD31^+^ SVF differed from unsorted SVF in certain functional properties such as ECM remodeling. The Ig‐family member CD31 and CD31^+^ cells, such as endothelial and immune cell subsets, have been shown to play a crucial role in regulating immune cell survival and homeostasis [65], particularly in controlling T‐cell trafficking and maintaining immune balance, mechanisms that are essential for modulating inflammatory processes [21, 22, 66, 67]. CD31^+^ cells have also been reported to promote anti‐inflammatory cytokine secretion, such as IL‐10 and IL‐1RA [20, 68]. In this study, CD31^+^ SVF exerted notable immunomodulatory effects by reducing TNF‐α and MCP‐1 expression in cartilage, suppressing *Ccl2* in the IFP, and attenuating PI3K‐Akt and MAPK signaling. Although CD31^+^ SVF and unsorted SVF demonstrated comparable anti‐inflammatory efficacy, transcriptomic analysis suggests that they may engage partially distinct molecular pathways to mediate these effects. Our previous study has shown that CD31^+^ cells enhance secretion of the anti‐inflammatory cytokine IL‐1RA (lower IL‐1α/IL‐1RA and IL‐1β/IL‐1RA ratios) and suppress pro‐inflammatory factors such as GM‐CSF and IFN‐γ [20]. Interestingly, these effects were significantly altered when monocytes were either enriched or depleted, emphasizing the importance of cellular composition when used therapeutically. Previous work demonstrated that SVF, in comparison with saline controls, significantly slowed OA progression and improved cartilage integrity through upregulation of COL2 and SOX9 and downregulation of MMP‐13 and ADAMTS‐5 [14]. The presence of M2 macrophages within SVF contributed to enhanced chondroprotection and joint homeostasis through the secretion of chondroprotective cytokines and growth factors [14]. In our study, CD31^+^ enrichment altered the SVF composition and may account for the distinct ECM remodeling profile, including lower levels of fibronectin, COL1A1, COL12A1, annexin, and tenascin XB. However, not all individual cartilage‐associated markers changed equally following CD31^+^ enrichment. For example, although ADAMTS4 protein reduction was observed more clearly in the SVF group than in the CD31^+^ SVF group, transcriptomic analysis showed that *Adamts4* was significantly downregulated in both treatment groups. This suggests that the non‐significant protein‐level result in the CD31^+^ SVF group may reflect incomplete concordance between transcriptional and protein‐level responses, rather than an absence of pathway regulation. Such selective divergence is consistent with our overall interpretation that CD31^+^ enrichment refines the therapeutic profile of SVF rather than enhancing every molecular readout to the same extent. Collectively, these findings suggest that CD31^+^ SVF may constitute a functionally relevant subpopulation of SVF with different immunomodulatory and cartilage‐protective mechanisms.

While there was no statistically significant difference in histopathological changes between SVF and CD31^+^ SVF, CD31^+^ SVF showed a more consistent pattern of tissue preservation than unfractionated SVF.

Beyond the anti‐inflammatory and chondroprotective effects, compared to the DPBS and SVF treatments, CD31^+^ SVF demonstrated partially superior efficacy on the modulation of subchondral bone remodeling, a critical contributor to OA progression that is molecularly and functionally interconnected with cartilage [37, 69]. The subchondral bone‐protective effects of CD31^+^ SVF were evidenced by significantly decreased BV/TV values in the medial subchondral plate, along with significant improvements in most trabecular parameters across both medial and lateral regions, compared to SVF and/or DPBS controls. The subchondral plate BV/TV changes differed slightly between the medial and lateral compartments. This reflects spatial heterogeneity of subchondral remodeling, likely related to compartment‐specific loading and baseline lesion severity, which is more pronounced medially in the spontaneous OA model similar to human KOA. This pattern is consistent with the recognized late‐OA sclerotic remodeling phenotype, in which abnormal mineralization and altered trabecular architecture coexist [37]. CD31^+^ cells have been shown to secrete immunoregulatory and osteoregulatory factors, including IL‐1RA, IL‐10, and VEGF [19, 20, 68], which may modulate osteoblast and osteoclast activity and promote microvascular remodeling [70, 71, 72]. Prior studies using CD31^+^ cell subsets derived from blood or adipose tissue demonstrated enhanced vascularization and reduced fibrosis in impaired bone healing and erectile dysfunction models, driven by combined angiogenic and anti‐inflammatory signaling [19, 20]. It is plausible that CD31^+^ enrichment within SVF preserves endothelial progenitors and anti‐inflammatory immune subsets capable of modulating subchondral bone dynamics via paracrine cytokine signaling. This may help restore microcirculation and rebalance bone remodeling. In support of this interpretation, CD31/EMCN co‐staining showed that CD31^hi^EMCN^hi^‐positive vascular‐associated structures at the osteochondral junction and peri‐osteophytic regions were most abundant in DPBS joints, reduced in SVF‐treated joints, and least prominent in CD31^+^ SVF‐treated joints. This pattern is consistent with attenuation of aberrant vascular‐associated remodeling at the osteochondral interface in OA. Together with the µCT outcomes, these findings support a vascular‐regulatory role of CD31^+^ SVF in osteochondral remodeling. Although the present study did not directly characterize the in vivo secretome of the injected cells, our previously published proteomic analysis of CD31^+^ SVF [19] identified several candidate secreted mediators enriched in the CD31^+^ fraction, including ANGPT2, DKK3, ANXA2, and VIM. An additional exploratory GO re‐analysis of this published secretome dataset [19] highlighted enriched patterns related to cell–matrix interaction and tissue microenvironment organization (Figure S3), providing complementary biological context for the remodeling‐related changes observed. Together with the reduced RANKL expression in cartilage, attenuation of osteochondral junction‐associated CD31^hi^EMCN^hi^‐positive vascular structures, and improved µCT parameters, these findings support a plausible concept in which CD31^+^ enrichment influences osteochondral homeostasis through paracrine vascular‐ and tissue‐regulatory effects. However, the specific mediators responsible for these effects remain to be directly tested in future studies. Notably, comparative GO enrichment analysis in this study revealed that CD31^+^ SVF and SVF modulate distinct biological processes such as ossification, suggesting mechanistic differences that may underlie the overall greater efficacy of CD31^+^ SVF in subchondral bone modulation. These findings suggest that the subchondral bone‐protective effects of CD31^+^ SVF may extend beyond SVF‐mediated mechanisms, with CD31^+^ cells representing a functionally distinct subpopulation that engages in osteochondral crosstalk through secreted factors. Further investigation into the cellular composition and secretome of CD31^+^ SVF could reveal novel therapeutic targets for optimizing osteochondral regeneration.

Several limitations of this study must be acknowledged. First, while our study demonstrated efficacy at the administered dose, a dose‐response study is warranted to identify the most effective and safe cell concentration. Second, our study utilized a single injection of SVF or CD31^+^ SVF, limiting our ability to assess whether multiple injections would provide superior or prolonged therapeutic effects. Third, the use of SVF derived from three independent donors may have introduced variability in the administered cell populations, potentially contributing to heterogeneity in the observed therapeutic outcomes in this study. Fourth, this study employed a xenogeneic design in which human‐derived SVF and CD31^+^ SVF were administered to immunocompetent guinea pigs. Although this approach was previously reported [73] and supported by our safety data showing no evidence of overt local or systemic immune reactions, subclinical xenogeneic host responses cannot be entirely excluded. Future studies should incorporate comprehensive cell phenotyping such as single‐cell RNA sequencing to correlate specific cell populations with therapeutic outcomes. In addition, direct comparison of SVF fractions enriched for other candidate surface markers would help determine whether CD31^+^ enrichment represents the therapeutically most relevant strategy for OA. This approach would not only enhance our understanding of the mechanisms underlying SVF‐mediated repair capacities but also facilitate the selection of the most potent and clinically relevant cell populations like CD31^+^ SVF for OA treatment.

In summary, both SVF and CD31^+^ SVF demonstrated significant therapeutic effects in experimental OA by mitigating inflammation, preserving cartilage structure, and attenuating subchondral bone remodeling. Notably, CD31^+^ SVF exhibited comparable anti‐inflammatory and chondroprotective efficacy to unsorted SVF, although these effects may be mediated through partially distinct molecular mechanisms. Moreover, compared to SVF, CD31^+^ SVF showed enhanced and consistent effects on regulating cartilage ECM remodeling and trabecular bone microarchitecture. From a clinical perspective, these findings suggest that CD31^+^ cell enrichment may provide a refined SVF treatment strategy for ECM preservation and osteochondral remodeling in OA. Rather than supporting immediate clinical implementation, the present animal data provide a rationale for further translational studies to determine the feasibility, safety, dosing, and therapeutic efficacy of this approach in patients with early‐to‐moderate KOA who remain insufficiently responsive to conservative treatment but are not yet candidates for joint replacement surgery.

## 4 Materials and Methods

### 4.1 Study Design and Experimental Procedures

A total of 40 DH guinea pigs (Envigo RMS GmbH, Germany) were used in this study: ten 2‐month‐old animals (five males and five females) were euthanized at 2 months and designated as a healthy control group, while 30 animals (15 males and 15 females) were included at the age of 6 months and randomly assigned to three experimental groups (*n* = 10 per group, five males and five females) (Figure 1A). Each experimental group, at 6 months of age, received a single 200 µL IA injection in both knee joints of either: DPBS or 1 × 10^6^ SVF in DPBS or CD31^+^ SVF in DPBS. Before IA injection, all experimental animals underwent in vivo MRI to assess baseline joint status. At this time point, no overt macroscopic OA lesions were detected by MRI, consistent with the early‐stage spontaneous OA phenotype of the DH guinea pig model at this age [26]. All injections were administered bilaterally under general anesthesia with isoflurane, followed by oral metamizole for 48 h for post‐procedural analgesia. Animals were monitored daily and housed in pairs under standard conditions throughout the 6‐month observation period. Tissue and imaging samples were collected at the study endpoint for further evaluation (Figure 1B). At the study endpoint, body weight analysis revealed no statistically significant differences between groups. All animal experiments complied with institutional guidelines and the German Laws for Animal Protection and Use. The study was approved by the local ethics committee on animal research (Landesamt für Gesundheit und Soziales Berlin, Germany, LaGeSo No. G0091/20). Approval for SVF preparation and application was also granted by the Danish Animal Experiments Inspectorate (Dyreforsøgstilsynet, No. 2022‐15‐0201‐01157).

### 4.2 Cell Isolation of SVF and CD31^+^ SVF

Human SVF was obtained from Blue Cell Therapeutics Ltd. (Denmark). SVF was derived from liposuctions of three donors. No separate approval was required for the collection of patient samples, according to the local Danish Science Ethics Committee (Videnskabsetisk Komité; agreement no. H‐22037018, 2022). Adipose tissue was collected during general anesthesia via water‐jet‐assisted liposuction and subsequently processed using the automated Celution 800/CRS system (Cytori Therapeutics, San Diego, CA, US), which employs enzymatic digestion to isolate SVF [74]. To isolate CD31^+^ cells from SVF, SVF with a concentration of 1 × 10^8^ cells/mL was incubated with a conjugated CD31/APC antibody (Blue Cell Therapeutics Ltd., Denmark) for 10 min. A PBS‐based MACSQuant Tyto running buffer (130‐107‐206, Miltenyi Biotec, Germany) was used for all washing steps and antibody incubations. The labeled SVF was then placed into a MACSQuant Tyto cartridge (130‐104‐791, Miltenyi Biotec, Germany), and CD31^+^ cells were sorted using a MACSQuant Tyto cell sorter (Miltenyi Biotec, Germany). The unsorted SVF and sorted CD31^+^ SVF fractions were stained with fluorophore‐conjugated antibodies, including CD31/APC, CD45 (130‐110‐638, Miltenyi Biotec, Germany) and CD90 (130‐114‐880, Miltenyi Biotec, Germany). Flow cytometric analysis was performed using a CytoFLEX LX Flow Cytometer (Beckman Coulter, US). Data were acquired using CytExpert software (Beckman Coulter, US) and analyzed using FlowJo software (version 10, BD Life Sciences, US).

### 4.3 In Vivo MRI Acquisition and Analysis

MRI was conducted on both knee joints of all animals. The healthy group underwent a single MRI scan at 2 months of age (endpoint measurement only). DPBS, SVF, and CD31^+^ SVF groups underwent MRI at 6 months (before IA injection), 7 months (1 month following IA injection), and 12 months of age (6 months following IA injection).

All images were acquired in vivo using a 3T MRI machine (MAGNETOM Skyra, Siemens, Germany) at the Department of Diagnostic and Interventional Radiology, Charité. Animals were anesthetized and positioned on a 3D‐printed anesthesia bed, ensuring secure immobilization of their hind legs in an extended position. To maintain body temperature, warm water‐filled gloves were placed next to the animals during imaging, and post‐scan recovery was conducted on a 37°C heat mat.

For MRI assessment, Sagittal Turbo‐spin‐echo (TSE) and 3D double‐echo (de3D) T2‐weighted image stacks of the knee joint were acquired. Healthy and DPBS groups were evaluated using a modified MOAKS scoring system, analyzing Hoffa synovitis and bone marrow lesions [27]. Joint tissues were assessed across all groups to evaluate post‐injection adverse reactions and joint integrity. All MRI assessments were conducted by two blind investigators with over 5 years of experience in OA research.

### 4.4 Histological, Immunohistochemical, and Immunofluorescence Analysis

Following euthanasia, left knee joints were carefully harvested and fixed in 4% paraformaldehyde for 48 h at room temperature, followed by decalcification in EDTA at 37°C. Specimens were dehydrated, embedded in paraffin, and sectioned at 6 µm thickness in the coronal plane. Following deparaffinization in xylene and rehydration with graded ethanol, sections were stained with Movat's pentachrome staining using alcian blue (8GS, Chroma, US), Weigert's haematoxylin (Merck, Germany), Brilliant Crocein/Acid Fuchsin (Brilliant Crocein R, Chroma, US, and Acid Fuchsin, Merck, Germany), 5% Phosphotungstic acid (Chroma, US), and Saffron du Gâtinais (Chroma, US) as previously described [75]. Moreover, H&E (Sigma–Aldrich, US), Safranin‐O (Carl Roth, Germany) and fast green (Thermo Fisher Scientific, US), and TB (Carl Roth, Germany) were conducted. Three non‐consecutive coronal sections among the central sections from each sample were stained with TB. Semi‐quantitative histopathological analysis was performed using the OARSI scoring system [26], with each section evaluated independently and blinded by two independent investigators, and an average score calculated per animal.

For immunohistochemical staining, sections were deparaffinized, rehydrated, and subjected to antigen retrieval using 0.05% trypsin or proteinase K. After blocking with 5% goat serum and 1% bovine serum albumin, primary antibodies targeting aggrecan (1:100) (PAB908Ra02, Cloud‐Clone, US), ADAMTS‐4 (1:100) (ab185722, Abcam, US), MMP‐13 (1:100) (PAA099Ra01, Cloud‐Clone, US), IL‐1β (1:100) (PAA563Gu01, Cloud‐Clone, US), MCP‐1 (1:100) (PAA087Gu01, Cloud‐Clone, US), and TNF‐α (1:150) (PAA133Gu01, Cloud‐Clone, US), and Periostin (1:100) (PAH339Ra01, Cloud‐Clone, US) were applied overnight at 4°C. Subsequently, secondary antibody staining (1:200) (goat anti‐rabbit, BA‐1000, Vector Laboratories, US) was performed. VECTASTAIN ABC‐AP Reagent (AK‐5000, Vector Laboratories, US) and ImmPACT Vector Red AP Substrate (SK‐5105, Vector Laboratories, US) were used for visualization, followed by nuclear counterstaining with methyl green (198080, Sigma–Aldrich, US). For RANKL staining, sections of the tibial subchondral bone region were deparaffinized, rehydrated, and processed for antigen retrieval. Sections were then incubated overnight at 4°C with a primary antibody against RANKL (1:150) (BA1323, Boster, China), followed by secondary antibody incubation and DAB‐based visualization. Nuclear counterstaining was performed with hematoxylin.

For COL1/COL2 double immunofluorescence staining, antigen retrieval was performed with citrate buffer (Citric acid monohydrate, C1909, and sodium citrate dihydrate, W302600, Sigma–Aldrich, US) and proteinase K (23NX.1, Carl Roth, Germany). Sections were incubated overnight with COL1 by targeting COL1A1 (1:50) (NBP1‐30054, Novus Biologicals, US) and COL2 by targeting COL2A1 (1:50) (MA5‐12789, Invitrogen, US) primary antibodies at 4°C overnight. The sections were washed and incubated with the secondary antibodies Alexa Fluor Plus 555 (1:400) (goat anti‐mouse, A32727, Invitrogen, US) and Alexa Fluor 405 (1:400) (goat anti‐rabbit, A31556, Invitrogen, US) and Sytox deep red (S11381, Invitrogen, US) nuclear counterstain. For CD31/EMCN double immunofluorescence staining, sections were subjected to antigen retrieval as described above and incubated overnight at 4°C with primary antibodies against CD31 (1:1000) (ab182981, Abcam, US) and EMCN (1:800) (ER1908‐09, HUABIO, US). After washing, the sections were incubated with fluorophore‐conjugated secondary detection reagents from the HISTOV multi‐label immunofluorescence kits, including DFT57100 (HISTOV, 1:1000) and DFT52100 (HISTOV, 1:1000), according to the manufacturer's instructions. Nuclei were counterstained with DAPI (WK00059, Wknow). Fluorescence images were acquired under identical exposure settings for all groups and used to assess the spatial distribution of CD31^hi^EMCN^hi^‐positive vascular‐associated structures in the osteochondral region.

Protein expression levels in the whole layer of the medial tibial cartilage were analyzed using a comprehensive digital pathology image analysis software Qupath, with intensity measurements quantified [76].

### 4.5 MALDI‐MSI and Data Analysis

Sample preparation for MALDI‐MSI was performed as previously described [77]. Briefly, formalin‐fixed paraffin‐embedded knee joints were sectioned at 4 µm thickness and mounted onto conductive glass slides (IntelliSlides, Bruker Daltonik GmbH, Germany). Paraffin was removed with xylene, followed by tissue rehydration in graded ethanol and distilled water. Trypsin/Lys‐C enzyme mix (25 µg/mL in 20 mM ammonium bicarbonate with 0.01% glycerol; Promega, Madison, WI, US) was homogeneously applied onto each slide using an automated sprayer (HTX TM‐Sprayer, HTX Technologies LLC, Chapel Hill, NC, US) under the following conditions: 16 layers, 0.015 mL/min flow rate, 750 mm/min spray velocity, 2 mm track spacing, and a nozzle temperature of 30°C. The enzymatic digestion was achieved by incubating the slides for 2 h at 50°C in a humidity chamber filled with saturated potassium sulfate solution. The slides were scanned using a digital slide scanner (Reflecta MF 5000; reflecta, Eutingen im Gäu, Germany). α‐cyano‐4‐hydroxycinnamic acid (CHCA) matrix (7 g/L in 70% acetonitrile, 1% trifluoroacetic acid (TFA); Bruker Daltonik GmbH) was applied using the HTX Sprayer. The spraying parameters were: 4 passes, 0.120 mL/min flow rate, 1200 mm/min velocity, 3 mm track spacing, and a nozzle temperature of 75°C. MALDI‐MSI was conducted on the rapifleX MALDI Tissuetyper (Bruker Daltonik GmbH) operated in positive ionization reflector mode (m/z 600–3200), using 500 laser shots per spot, 1.25 GS/s sampling rate, and 50 µm raster width. Data were acquired with FlexImaging 6.0 and flexControl 4.2 software (Bruker Daltonik GmbH). External calibration was performed using an on‐slide standard (Peptide Calibration Standard II, Bruker Daltonik GmbH). Post‐acquisition, the matrix was removed with 70% ethanol, sections were stained with SO‐FG and scanned at 20× magnification (NanoZoomer‐SQ, Hamamatsu Photonics K.K, Hamamatsu City, Japan).

MALDI‐MSI raw data were processed using SCiLS Lab 2024a Pro software (Bruker Daltonik GmbH). Baseline removal was performed using the TopHat algorithm (peak width: 200), followed by total ion count (TIC) normalization. Peak detection and alignment were conducted using a standard segmentation pipeline, with TIC normalization, no denoising, and a minimal peak interval of ± 0.111 Da. Region of interest (ROI), defined as the superficial 30% of the medial tibial plateau cartilage based on SO‐FG staining, was selected for analysis. ROC curve and Wilcoxon rank‐sum test analyses were performed to identify discriminative MALDI‐MSI m/z values. The analyses were conducted on all individual spectra from a randomized subset of the dataset, matched to the minimum number of spectra common to both comparison groups. For ROC analysis, the AUC ranges from 0 to 1, with values approaching 0 or 1 indicating strong discriminatory potential, and a value of 0.5 indicating no discrimination. Peptides with an AUC > 0.6 or < 0.4 and *p* value < 0.05 were considered candidate markers.

### 4.6 Protein Identification by Nano‐LC‐ESI‐MS/MS

Bottom‐up nano‐liquid chromatography electrospray ionization tandem mass spectrometry (nano‐LC‐ESI‐MS/MS) was performed on serial tissue sections to identify m/z features detected by MALDI‐MSI. Slides were processed according to the MALDI‐MSI protocol up to enzymatic digestion. Peptides were then extracted from the tissue surface with 100 µL of 0.1% TFA at room temperature, purified with ZipTip C18 pipette tips (Merck KGaA, Darmstadt, Germany) following the manufacturer's protocol, dried by vacuum centrifugation, reconstituted in 20 µL of 0.1% TFA, and stored at −20°C until analysis. For nano‐LC‐ESI‐MS/MS, 4 µL of peptide eluate was injected into a Dionex Ultimate 3000 nano‐HPLC system (Thermo Fisher Scientific) coupled to a timsTOF HT fleX mass spectrometer (Bruker Daltonik GmbH, Bremen, Germany) equipped with a CaptiveSpray ESI source. Peptides were first loaded in 0.1% TFA onto an Acclaim PepMap 100 C18 trap column (2 cm × 100 µm × 5 µm, Thermo Fisher Scientific), then separated on an Aurora ultimate CSI C18 analytical column (25 cm × 75 µm × 1.7 µm; Bruker Daltonik GmbH) using a 60‐min gradient from 3% to 37% ACN in 0.1% formic acid. The flow rate was 400 nL/min, the column temperature 60 °C, and the system operated within a pressure range of 10 to 800 bar.

Mass and ion mobility calibration were performed using ESI‐L tuning mix (Agilent Technologies, Inc., Santa Clara, California, US), applying enhanced quadratic (mass) and linear (ion mobility) calibration modes. Data acquisition was carried out using the DDA‐PASEF method with TIMS enabled. The mass spectrometer operated in a mass range of m/z 100–1700, with an ion mobility window of 0.85–1.30 Vs/cm^2^, and a TIMS ramp time of 100 ms. Precursor ions were fragmented via collision‐induced dissociation. Raw data (.baf) were analyzed in PEAKS Studio 11.5 (Bioinformatics Solutions Inc., Waterloo, ON, Canada) [78], searching against the UniProtKB/Swiss‐Prot Cavia porcellus database (downloaded 23.10.2024) with 20 ppm precursor and 0.05 Da fragment mass tolerance, trypsin as an enzyme (2 missed cleavages), and up to 7 variable PTMs including methionine oxidation, proline/lysine hydroxylation, and N‐terminal acetylation. FDR was set at 1%, with identification requiring ≥ 1 unique peptide. Only the top‐scoring ID per protein group was retained. Proteins matched to MALDI‐MSI features required ≥ 2 peptides with consistent spatial distribution [77, 79].

### 4.7 Serum Cytokine Analysis by ELISA

Blood samples from the DPBS, SVF, and CD31^+^ SVF groups were collected, clotted for 20 min, and centrifuged at 3500 rpm for 10 min at room temperature. The supernatant serum was collected and stored at – 80°C until analysis. Multiplex enzyme‐linked immunosorbent assay (ELISA) investigating inflammatory profile was performed using the Bio‐Plex Pro Mouse Cytokine 23‐plex Assay (M60009RDPD, US) and Bio‐Plex 200 system (Bio‐Rad, US). Serum C_3_M was analyzed at the Nordic Biosciences lab using automated CLIA on the i10 platform. Manual ELISA was measured in duplicates and automated assays were measured in singles. All analyses were performed according to the manufacturer's protocol.

### 4.8 RNA Extraction and RT‐qPCR Analysis

Cartilage and IFP tissues were harvested from the right knee joints of all animals, snap‐frozen, and stored at ‐80°C until further processing. Samples were then homogenized, and total RNA was extracted. Reverse transcription of total RNA into complementary DNA (cDNA) was performed using a Reverse Transcription Kit (K1691, Thermo Fisher Scientific, US) with Oligo(dT)_18_ primers (10753741, Thermo Fisher Scientific, US) following the manufacturer's instructions. Subsequently, RT‐qPCR was conducted using PowerTrack SYBR Green Master Mix (A46113, Applied Biosystems, US) on a QuantStudio 5 Real‐Time‐PCR‐Systeme (Thermo Fisher Scientific, US). Glyceraldehyde‐3‐phosphate dehydrogenase (GAPDH) served as the housekeeping gene for normalization.

Two normalization strategies were applied for comparative gene expression analysis. First, the mRNA levels in the healthy and DPBS groups were normalized against the healthy group to evaluate disease‐related changes. Secondly, the mRNA expression levels in the DPBS, SVF and CD31^+^ SVF groups were normalized to the DPBS group to evaluate the effects of treatments. The primer sequences used for amplification are listed in Table S5.

### 4.9 mRNA‐seq and Data Analysis

For mRNA sequencing (mRNA‐seq) analysis, total RNA of cartilage (*n* = 4 per group) and IFP (*n* = 4 per group) with RNA integrity numbers (RIN) > 7.0 were sent for library preparation. RNA quantification and quality assessment were performed using Qubit fluorometry and the Agilent TapeStation system. Library preparation and mRNA‐seq were conducted by the Genomics Technology Platform at the Berlin Institute of Health (BIH), Charité. Data analysis was performed by the Core Unit Bioinformatics at the BIH, Charité. Libraries were sequenced on the Illumina NovaSeq X Plus platform to generate paired‐end 150 bp reads with an average of ∼50 million reads per sample. cDNA libraries were generated using the Illumina TruSeq RNA Sample Preparation Kit following the manufacturer's protocol. Sequencing was performed on an Illumina NovaSeq X Plus platform to obtain paired‐end reads (2 × 150 bp) with an average of 50 million reads per sample.

Raw FASTQ data underwent standard quality control steps using FastQC, Dupradar, Qualimap, RNA_seqc and Preseq to assess read quality, followed by adapter and quality trimming with fastp. Low‐quality bases (Q < 20) and reads shorter than 36 bp were removed to ensure high‐quality data for subsequent analysis. Cleaned reads were aligned to the Cavia porcellus (guinea pig) reference genome (assembly Cavpor3.0, GCA_000151735.1) using the STAR aligner, v.2.7.11a [80]. The corresponding GTF annotation file was used to guide the alignment. Raw gene expression counts were generated using featureCounts, which assigned uniquely mapped reads to annotated genomic features.

For purposes of PCA and visualization, raw counts were normalized using the Regularized Log Transformation (RLD) implemented in the DESeq2 R package [81], version 1.38. PCA was then performed on the RLD‐transformed data to visualize global gene expression differences between the experimental groups.

Paired comparisons between groups were conducted using DESeq2, with DEGs defined as those with an adj. *P* < 0.05 and an |log_2_FC| > 1. DEGs were used to generate hierarchical clustering heatmaps based on their RLD values, enabling visualization of group‐specific expression patterns. Pairwise comparisons included SVF vs. DPBS, CD31^+^ SVF vs. DPBS, and CD31^+^ SVF vs. SVF. Volcano plots were generated to visualize the DEG results, and key OA‐related genes were annotated on the plots. GO enrichment analysis was performed on the DEGs from pairwise comparisons to identify overrepresented biological processes. KEGG pathway analysis was conducted to determine significantly enriched signaling pathways.

### 4.10 µCT Scanning and Subchondral Bone Analysis

Before decalcification, left knee joints were scanned using a µCT scanner (Skyscan 1172, Bruker, MA, US) with a 12.95 µm pixel resolution, 0.5‐mm aluminum filter, and 70 kV/142 µA settings. 3D reconstructions were performed using NRecon software as previously reported [82]. Both medial and lateral subchondral bone analysis was conducted using ImageJ (NIH).

A load‐bearing region with an area of 2.0 × 2.0 mm^2^ was defined as the ROI for the subchondral plate. Additionally, a cuboid volume of 2.0 × 2.0 × 0.8 mm^3^, located beneath the subchondral plate, was selected as the volume of interest for the trabecular bone.

### 4.11 Statistical Analysis

Statistical analyses were performed using SPSS version 29.0.1 (IBM SPSS Statistics, Chicago, IL, US). Data are presented as mean ± standard error of the mean (SEM), unless otherwise indicated. Data ranges and 95% confidence intervals (CIs) were additionally reported for primary outcome measures where applicable. All tests were two‐sided. For comparisons among multiple groups, two‐way analysis of variance (ANOVA) was used, as appropriate, followed by Tukey's post hoc test for pairwise comparisons. For comparisons between two groups, a Paired Sample T‐Test was used. Statistical significance was defined as ^*^ *p* < 0.05, ^**^ *p* < 0.01, and ^***^ *p* < 0.001, unless otherwise stated.

## Author Contributions

Study design: T.M, S.J.Z, and T.W. Methodology: S.J.Z., T.W., B.C., M.G., F.N.F., D.G., A.H., D.A., M.A.M., B.C., J.W.3. Data collection: S.J.Z., T.W., B.C., M.G., Y.Y.L., D.A., Validation: T.W., S.G., G.N.D, S.P.S. O.K., T.M. Manuscript Drafting: S.J.Z., M.G., Y.Y.L., T.M. Review & editing: T.W., Y.Y.L., J.W.3., S.G., G.N.D., S.P.S., O.K., T.M. All authors contributed to the article and approved the submitted version.

## Funding

This work was supported by a Grand Solutions Grant from Innovation Foundation Denmark under grant agreement no. 7051. Further funding was provided by the Federal Ministry of Education and Research (BMBF) under grant agreement no. 031L0234B, the European Union under grant agreement no. 101095635 (PROTO), and the German Research Foundation (DFG) through the Collaborative‐Research‐Center‐1444. A.H. is supported by the BIH Charité Junior Clinician Scientist Program, which is funded by Charité – Universitätsmedizin Berlin and the Berlin Institute of Health at Charité. Views and opinions expressed are those of the authors only and do not necessarily reflect those of the European Union or the European Health and Digital Executive Agency (HADEA). Neither the European Union nor the granting authority can be held responsible for them. The funders had no role in study design, data collection and analysis, decision to publish, or preparation of the manuscript.

## Consent for Publication

No written consent for publication was required, as no patient identifiable information is included in this study.

## Conflicts of Interest

The authors declare no conflicts of interest.

## Supporting information




**Supporting File 1**: advs76187‐sup‐0001‐FiguresS1‐S5.docx.

**Supporting File 2**: advs76187‐sup‐0002‐TableS1‐S5.xlsx.

**Supporting File 3**: advs76187‐sup‐0003‐TableS6.xlsx.

## Data Availability

The data that support the findings of this study are available from the corresponding author upon reasonable request.
