# Supplementary material for: CD31+ Cell Enrichment Enhances Therapeutic Effects of Stromal Vascular Fraction in Experimental Primary Osteoarthritis: A Preclinical Study in the Dunkin Hartley Guinea Pig Model
Source: Adv Sci (Weinh). 2026 Jul 11;13(51):e76187. doi: 10.1002/advs.76187 (PMC13355895; doi:10.1002/advs.76187)
Supplement: Supplementary file 1 [file ADVS-13-e76187-s001.docx]

**Supplementary Figure 1. Histological confirmation of early-stage spontaneous osteoarthritis in 6-month-old Dunkin Hartley guinea pigs.** (A) Representative histological images of the medial tibial cartilage from 6-month-old Dunkin Hartley (DH) guinea pigs stained with Hematoxylin and eosin (H&E) and Toluidine blue (TB). These images show mild but detectable OA-related alterations, including early surface irregularity and reduced proteoglycan staining, consistent with early-stage spontaneous OA in this model. (B) Semi-quantitative histopathological evaluation of the 6-month-old DH guinea pig knee joints using the OARSI scoring system, including cartilage structure, proteoglycan content, osteophyte formation, and total histological score. Individual data points are presented as means ± SEM.

**Supplementary Figure 2. Protein-level validation of POSTN, RANKL, and CD31/EMCN-associated changes in OA joints following SVF and CD31⁺ SVF treatment.** (A) Representative images of POSTN and RANKL staining in medial tibial cartilage and peri-osteophytic regions of DPBS-, SVF-, and CD31⁺ SVF-treated joints. Dashed boxes indicate magnified regions. (B) Quantification of POSTN and RANKL expression by integrated optical density (IOD/μm²). Individual data points are shown together with mean ± SEM. Statistical comparisons were performed using two-way ANOVA with Tukey’s HSD post hoc test. **P* < 0.05, ***P* < 0.01, and ****P* < 0.001. (C) Representative double immunofluorescence staining for EMCN (green), CD31 (red), DAPI (blue), and merged images in the osteochondral region of DPBS-, SVF-, and CD31⁺ SVF-treated joints. CD31^hi^EMCN^hi^-positive vascular-associated structures were more frequently observed in the DPBS group, reduced in the SVF group, and least prominent in the CD31⁺ SVF group. Individual data points are presented as means ± SEM (A-C and E-G). Statistical comparisons were performed using two-way ANOVA with Tukey’s post hoc test. **P* < 0.05, ***P* < 0.01, and ****P* < 0.001.

**Supplementary Figure 3. GO enrichment analysis of upregulated proteins in the previously published CD31⁺ secretome dataset.** GO enrichment analysis of proteins previously identified as upregulated in the CD31⁺ fraction relative to the CD31⁻ fraction in our published proteomic dataset (19). Shown are the enriched GO terms in the Biological Process, Cellular Component, and Molecular Function categories. Bubble size indicates gene count, and color indicates adjusted *P* value.

**Supplementary Figure 4. Expanded healthy-reference analysis of MMP-13 protein expression and cartilage-associated gene expression in the DH guinea pig OA model. (A)** Representative immunohistochemical staining of MMP-13 in medial tibial cartilage from the 2-month-old Healthy group and quantitative comparison of MMP-13 expression across Healthy, DPBS, SVF, and CD31⁺ SVF groups. Representative images of the treatment groups are shown in the corresponding main figure. **(B–E)** RT-qPCR analysis of cartilage-associated genes in medial tibial cartilage across the four groups. For qPCR analysis, gene expression values were normalized to the sex-matched Healthy reference, with male and female samples normalized separately to the mean value of their corresponding Healthy subgroup. The Healthy group serves as a young pre-disease reference while preserving individual biological variation within each sex. Because 2-month-old DH guinea pigs are still skeletally immature, differences relative to the 12-month groups may reflect both developmental maturation and OA-related pathological changes. Data are presented as individual data points with mean ± SEM. Statistical comparisons were performed using two-way ANOVA with Tukey’s post hoc test. Asterisks shown directly above the DPBS, SVF, and CD31⁺ SVF bars in panels B–E indicate comparisons versus the corresponding sex-matched Healthy reference. **P* < 0.05, ***P* < 0.01, and ****P* < 0.001.

**Supplementary Figure 5. Expanded healthy-reference comparison of cartilage MCP-1 protein expression and IFP inflammatory gene expression in the DH guinea pig OA model.** (**A**) Representative immunohistochemical image of MCP-1 staining in medial tibial cartilage from the 2-month-old healthy group and quantitative comparison of MCP-1 expression across Healthy, DPBS, SVF, and CD31⁺ SVF groups. Representative images for the DPBS, SVF, and CD31⁺ SVF groups are shown in the corresponding main figure. (**B**) RT-qPCR analysis of inflammatory markers in the infrapatellar fat pad (IFP) across Healthy, DPBS, SVF, and CD31⁺ SVF groups. For qPCR analysis, gene expression values were normalized to the sex-matched Healthy reference, with male and female samples normalized separately to the mean value of their corresponding Healthy subgroup. The Healthy group serves as a young pre-disease reference while preserving individual biological variation within each sex. Because 2-month-old DH guinea pigs are still skeletally immature, differences relative to the 12-month groups may reflect both developmental maturation and OA-related pathological changes. Data are presented as individual data points with mean ± SEM. Statistical comparisons were performed using two-way ANOVA with Tukey’s post hoc test. Asterisks shown directly above the DPBS, SVF, and CD31⁺ SVF bars in panel B indicate comparisons versus the corresponding sex-matched Healthy reference. **P* < 0.05, ***P* < 0.01, and ****P* < 0.001.
